# [^18^F]FDG PET/CT to reduce the need for sentinel lymph node biopsy in early-stage oral cancer: PETN0-study protocol

**DOI:** 10.1371/journal.pone.0325032

**Published:** 2025-07-01

**Authors:** Roosmarijn S. Tellman, Dominique N.V. Donders, Anne I. J. Arens, Koos Boeve, Adrienne H. Brouwers, Simone E.J. Eerenstein, Sylvia L. van Egmond, Thomas J.W. Klein Nulent, W. Martin C. Klop, Martin Lacko, Jochem A.J. van der Pol, Daphne D.D. Rietbergen, Robert P. Takes, Joris Tim, Wouter V. Vogel, Gerben J.C. Zwezerijnen, Bart de Keizer, Remco de Bree

**Affiliations:** 1 Department of Head and Neck Surgical Oncology, University Medical Center Utrecht, Utrecht, The Netherlands; 2 Department of Medical imaging, Radboud University Medical Center, Nijmegen, The Netherlands; 3 Department of Oral and Maxillofacial Surgery, University of Groningen, University Medical Center Groningen, Groningen, The Netherlands; 4 Department of Nuclear Medicine and Molecular Imaging, University of Groningen, University Medical Center Groningen, Groningen, The Netherlands; 5 Department of Otolaryngology and Head and Neck Surgical Oncology, Amsterdam University Medical Center location Vrije Universiteit Amsterdam, Amsterdam, The Netherlands; 6 Department of Otolaryngology and Head and Neck Surgical Oncology, Leiden University Medical Center, Leiden, The Netherlands; 7 Department of Oral and Maxillofacial Surgery and Head and Neck Surgical Oncology, Haaglanden Medical Center, The Hague, The Netherlands; 8 Department of Head and Neck Surgery, The Netherlands Cancer Institute-Antoni van Leeuwenhoek, Amsterdam, The Netherlands; 9 Department of Otorhinolaryngology and Head and Neck Surgical Oncology, Maastricht University Medical Center+, Maastricht, The Netherlands; 10 Department of Nuclear Medicine, Maastricht University Medical Center+, Maastricht, The Netherlands; 11 Department of Radiology & Nuclear Medicine, Leiden University Medical Center, Leiden, The Netherlands; 12 Department Otorhinolaryngology and Head and Neck Surgery, Radboud University Medical Center, Nijmegen, The Netherlands; 13 Department of Nuclear Medicine, Haaglanden Medical Center, The Hague, The Netherlands; 14 Department of Nuclear Medicine, The Netherlands Cancer Institute-Antoni van Leeuwenhoek, Amsterdam, The Netherlands; 15 Department of Radiology & Nuclear Medicine, Amsterdam University Medical Center location Vrije Universiteit Amsterdam, Amsterdam, The Netherlands; 16 Department of Radiology & Nuclear Medicine, University Medical Center Utrecht, The Netherlands; PLOS: Public Library of Science, UNITED KINGDOM OF GREAT BRITAIN AND NORTHERN IRELAND

## Abstract

Using reliable techniques for detecting lymph node metastases (LNM) in oral squamous cell carcinoma (OSCC) is crucial for adequate neck treatment. Currently, palpation of the neck, computed tomography, magnetic resonance imaging, ultrasound-guided fine needle aspiration cytology and/or sentinel lymph node biopsy (SLNB) are used to stage the neck in early-stage OSCC. SLNB is a reliable diagnostic technique to detect occult LNM. However, management of the neck with SLNB has its limitations. First of all, SLNB is an invasive procedure with associated morbidity and approximately 20–30% of patients require a subsequent neck dissection. Moreover, performing a subsequent neck dissection is more complex than elective neck dissection, and carries a higher risk of complications. Therefore, it is important to improve patient selection for SLNB. Fluor-18-fluorodeoxyglucose ([^18^F]FDG) positron emission tomography/computed tomography (PET/CT) has shown promising results for LNM detection. The aim of the PETN0 study, a prospective Dutch multicenter cohort study (registration number NL83442.041.22), is to reduce the need for SLNB by developing scoring criteria for [^18^F]FDG PET/CT with a high positive predictive value (PPV) in patients with early-stage OSCC. Developing scoring criteria for a high PPV can reduce SLNBs and second-stage neck dissections by performing a neck dissection together with resection of the primary tumor in patients with predicted LNM. When focused on high PPV the sensitivity will probably be lower, but missed LNM will be detected by SLNB when performed after negative [^18^F]FDG PET/CT. Patients (n = 159) with cT1-3N0 OSCC (8^th^ TNM edition; only when T3 is assessed based on tumor dimensions of >2 and ≤4 cm, with DOI > 10 mm), candidate for transoral excision and SLNB, are included in the study. [^18^F]FDG PET/CT will be conducted within a maximum of three weeks before SLNB. A cost-effectiveness analysis will also be performed, together with quality of life assessment using questionnaires.

## Introduction

In head and neck squamous cell carcinoma, it is crucial to reliably detect (occult) lymph node metastases because of the availability of adequate treatments of the neck, i.e. neck dissection and/or radiotherapy. Among available diagnostic modalities, sentinel lymph node biopsy (SLNB) has proven to be a reliable procedure to detect occult lymph node metastases in early-stage oral squamous cell carcinoma (OSCC) with a sensitivity of 87%, and negative predictive value of 94% [1]. As a result, SLNB is included in numerous national guidelines, including those from The Netherlands, United Kingdom (National Institute for Health and Care Excellence), and United States of America (National Comprehensive Cancer Network) [1–3]. However, management of the neck based on SLNB has its limitations. SLNB remains an invasive surgical procedure with associated morbidity, and approximately 20−30% of patients require a subsequent therapeutic neck dissection, which must be performed promptly in a separate operation, presenting potential logistical challenges [4,5]. Additionally, performing a subsequent neck dissection is more complex than elective neck dissection, and carries a higher risk of complications [3]. Moreover, a neck dissection as a second surgical procedure likely imposes a greater burden on patients and the healthcare system and incurs higher costs. Therefore, it is important to further improve patient selection for SLNB by investigating other (less invasive) diagnostics techniques such as Fluor-18-fluorodeoxyglucose ([^18^F]FDG) positron emission tomography/computed tomography (PET/CT).

Despite promising results, the role of [^18^F]FDG PET/CT to detect (occult) lymph node metastases in head and neck squamous cell carcinoma patients remains unclear. Comparing available studies is challenging due to variations in scan protocols, definitions of the clinically negative neck (cN0), criteria for PET positivity, scan reading methods, and reference standards [7]. Since PET imaging has improved considerably by integration of PET and CT devices, developments in detector systems yielding a higher image resolution, and optimization of head and neck acquisition parameters, new prospective studies are warranted [6].

The number of SLNB and second-stage subsequent completed neck dissection could be reduced by performing a neck dissection in the same operation as resection of the primary tumor in patients in whom occult lymph node metastases can be predicted with high positive predictive value (PPV) using [^18^F]FDG PET/CT. By varying cut-off levels of [^18^F]FDG uptake, scoring criteria can be developed and optimized to predict the presence of lymph node metastases with high PPV rather than high sensitivity, which is the usual focus in existing studies [2]. When focused on high PPV the sensitivity will probably be lower, but missed lymph node metastases will be detected by SLNB when performed after negative [^18^F]FDG PET/CT.

## Materials and methods

The prospective Dutch multicenter cohort study, in short: PETN0 study, is registered in the Netherlands Trial Register (NTR number NL83442.041.22) and approved by the Medical Ethics Committee NedMec (METC number 23–103/G). The study started in January 2024, and is conducted in eight head and neck centers of the Dutch Head and Neck Society: University Medical Center Utrecht, Amsterdam University Medical Center location Vrije Universiteit Amsterdam, Haaglanden Medical Center, Leiden University Medical Center, Maastricht University Medical Center + , The Netherlands Cancer Institute-Antoni van Leeuwenhoek, Radboud University Medical Center and University Medical Center Groningen. It is expected that recruitment is completed in January 2027, data collection is completed in January 2028 and results are available in April 2028.

Inclusion and exclusion criteria are presented in Table 1. Patients, diagnosed with early-stage OSCC and candidate for transoral excision and SLNB, who meet the criteria (Table 1) are asked to participate in the study. We aim to include 159 participants.

**Table 1:** Inclusion and exclusion criteria.

Inclusion criteria	Exclusion criteria
Early-stage OSCC ^†^ is defined as clinically T1-3^‡^, N0.	The patient has a history of treatment of the neck (neck dissection and/or radiotherapy).
The patient is ≥ 18 years of age at the time of consent.	The patient has US-FNAC positive for lymph node metastasis.
The patient has no palpable lymph nodes in the neck.	The patient has poorly controlled diabetes mellitus
The patient is a candidate for transoral excision and SLNB.	The patient refused preoperative imaging workups.
The patient has provided written informed consent authorization before participating in the study.	The primary tumor has a different pathology than squamous cell carcinoma.
Clinical nodal staging (cN0) has been confirmed by ultrasound, CT, and/or MRI if performed (not mandatory).	
Patients with prior malignancy of the head and neck area are allowed, provided the patient meets both of the following criteria: a. Underwent potentially curative therapy for all prior head and neck malignancies and is deemed low risk for recurrence; and b. No head and neck malignancy for the past three years and no evidence of recurrence.	

† Tumor location will be limited to the intraoral areas of mucosal lip, buccal mucosa, lower alveolar ridge, upper alveolar ridge, retromolar gingiva, retromolar trigone, floor-of-the-mouth, hard palate, and the mobile portion of the oral tongue. ‡ Only when T3 is assessed based on tumor dimensions of >2 cm and ≤4 cm with DOI > 10 mm (based on Tumor Nodal Metastasis (TNM) Staging 8^th^ Edition).

### Study procedure

**Fig 1:**
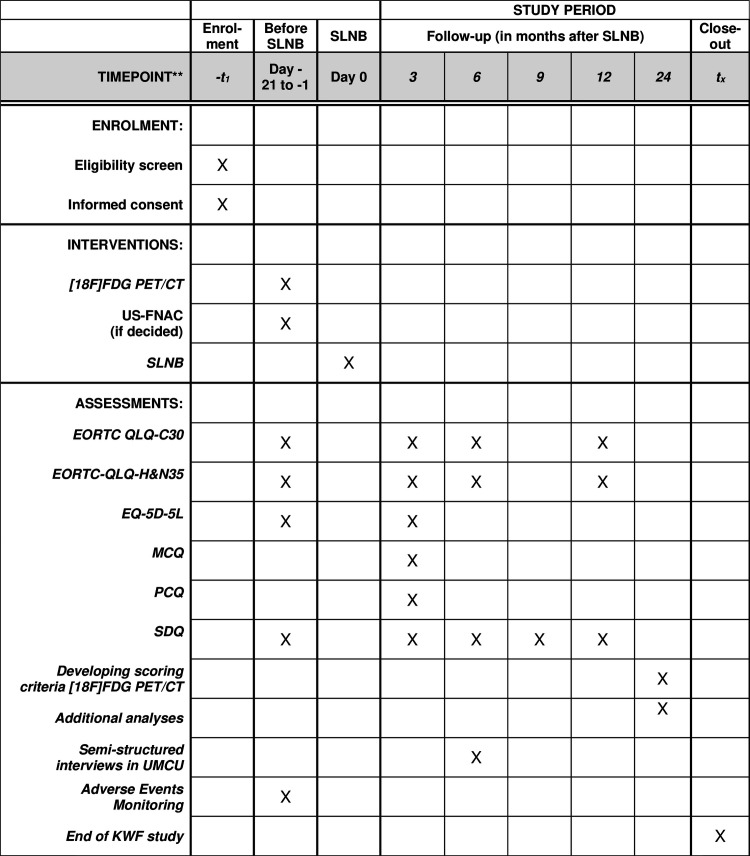
SPIRIT schedule of enrolment, interventions and assessments. SLNB = Sentinel Lymph Node Biospy, [^18^F]FDG = Fluor-18-fluorodeoxyglucose, PET = Positron Emission Tomography, CT = Computed Tomography, US-FNAC = Ultrasound Guided Fine Needle Aspiration Cytology, EORTC-QLQ-C30 = European Organization for Research and Treatment for Cancer Quality of Life Questionnaire Core 30, EORTC-QLQ-H&N35 = European Organization for Research and Treatment for Cancer Quality of Life Questionnaire Head & Neck 35, EQ-5D-5L = EuroQol Five-Dimensional Five-Level Questionnaire, MCQ = Medical Consumption Questionnaire, PCQ = Productivity Cost Questionnaire, SDQ = Shoulder Disability Questionnaire, UMCU = University Medical Center Utrecht, KWF = Koningin Wilhelmina Fonds Dutch Cancer Society.

The diagnostic intervention of this study is an [^18^F]FDG PET/CT and a contrast enhanced-CT of the neck within a maximum of three weeks before SLNB and questionnaires at five time points (Fig 1). Efforts will be made to perform the [^18^F]FDG PET/CT and SLNB within a one-week timeframe. The overall study matrix is presented in Fig 1.

### [^18^F]FDG PET/CT and contrast-enhanced CT

The [^18^F]FDG PET/CT will be acquired using European Association of Nuclear Medicine (EANM) Research Ltd. (EARL2) accredited PET/CT systems [7]. Patient preparation, scanner calibration, image acquisition, and reconstruction will be performed according to EANM standards [8,9]. After a fasting period of at least 6 hours, patients receive an intravenous injection of [^18^F]FDG. Approximately 60 minutes after the administration of the tracer, a PET/low-dose CT of the head and neck, as well as the chest, will be acquired, followed by a contrast-enhanced CT of the neck. If the patient has experienced a previous allergic reaction after the administration of contrast fluid, only a PET/low-dose CT of the head and neck and chest is sufficient for participation in this study and a contrast-enhanced CT of the neck will be omitted. To avoid false-positive results by cytological puncture, efforts will be made to perform the [^18^F]FDG PET/CT before ultrasound-guided fine needle aspiration cytology (US-FNAC). Patients with positive US-FNAC will be excluded from further analysis, as the neck is no longer clinically negative. Based on a prevalence of 27% for (occult) lymph node metastases detected with SLNB in early-stage OSCC [10] and a sensitivity of 15.4% for detecting occult lymph node metastases with US-FNAC (unpublished data, manuscript in preparation), it is estimated that approximately 4.2% (27% x 15.4%) of the participants in this study will be excluded due to a positive US-FNAC result.

### Sentinel lymph node biopsy

The SLNB will be performed according to current clinical practice and consensus guidelines [11–13]. Patients will undergo lymphoscintigraphy (including single photon computed tomography and CT) at 2 hours after peritumoral injection of technetium-99m-labeled nanocolloid, bound or not bound to indocyanine green, the day before surgery or the day of surgery. The SLNB will be blinded for the [^18^F]FDG PET/CT and contrast-enhanced CT scan. Therefore, the results of the [^18^F]FDG PET/CT and contrast-enhanced CT scan will not be used to alter surgical planning.

In case of a positive SLNB followed by a neck dissection, the surgeon will aim to identify and harvest [^18^F]FDG-positive lymph nodes separately from the neck dissection specimen, based on the anatomical findings of the [^18^F]FDG PET/CT scan. Identified [^18^F]FDG-positive lymph nodes will be sent to the pathologist for examination through step-serial sectioning and immunohistochemistry with HE and keratin staining, as is standard practice for sentinel lymph nodes [14]. All patients will be observed during 12 month follow-up in accordance with national guidelines [12]. Additionally, included patients will be asked for participation in a long-term follow-up study, with at least 24 months follow-up, to assess late regional recurrences and improve the reference standard even further.

### Outcomes

The primary objective of this study is to reduce the need for SLNB by [^18^F]FDG PET/CT in cN0 OSCC patients. Secondary objectives of this study are listed in Table 2.

**Table 2:** Primary and secondary objectives.

Primary objective	Secondary objectives
Reduce the need for SLNB by [^18^F]FDG PET/CT in cN0 OSCC patients	To optimize scoring criteria for the detection of (occult) lymph node metastases by [^18^F]FDG PET/CT with a high PPV.
	To assess the sensitivity, specificity, PPV, NPV and accuracy for the different scoring criteria.
	To investigate inter-observer agreement before and after scoring criteria are established.
	To compare the quality of life and costs in three different diagnostic scenarios 1) PET/CT, 2) SLNB, and 3) PET/CT and, only if negative, SLNB.
	To obtain insight into patients’ preferences and experience of the diagnostic procedures.

SLNB = Sentinel Lymph Node Biopsy, cN0 = clinically node-negative, OSCC = Oral Squamous Cell Carcinoma, PPV = Positive Predictive Value, NPV = Negative Predictive Value.

In the clinical setting, institutional nuclear physicians will only score the [^18^F]FDG PET/CT scan on relevant additional findings in the thorax and upper abdomen (e.g., distant metastases and second primary tumors). The effect of additional findings on a treatment plan and intent based on multidisciplinary team conference decisions will be analyzed.

Outside the clinical setting (after the SLNB) all [^18^F]FDG PET/CT and contrast-enhanced CT scans will be collected and individually scored by a panel of 5 experienced nuclear physicians. [^18^F]FDG PET/CT scans are scored using different parameters and cut-off values. All [^18^F]FDG-positive lymph nodes will be scored. The criteria for scoring [^18^F]FDG-positive lymph nodes, used by the panel of nuclear physicians, are detailed in Table 3.

**Table 3:** Scoring criteria [^18^F]FDG PET/CT.

Scoring criteria	
*Lymph nodes with [* ^*18*^ *F]FDG uptake exceeding normal physiological uptake*	Positive/ suspicious for lymph node metastasis.
*Visual scoring using a 5-point Likert scale*	1. completely negative; 2. probably negative; 3. equivocal; 4. probably positive; 5. positive based on the local surrounding background.
*The Hopkins criteria*	1. uptake less than IJV; 2. focal uptake greater than IJV but less than liver; 3. diffuse uptake greater than IJV or liver; 4. focal uptake greater than liver; 5. intense uptake.
*Semi-quantitative scoring of nodes with [* ^*18*^ *F]FDG uptake exceeding normal physiological uptake*	Manual volume of interest, SUV calculations are performed using the lean body mass formula.
*Morphology of lymph nodes >3mm*	Maximal and minimal axial diameter, maximum longitudinal/short axis diameter ratio, non-fat low density, and shape (spheric or round).

[^18^F]FDG = Fluor-18-fluorodeoxyglucose, IJV = Internal Jugular Vein, SUV = Standardized Uptake Value.

Quality of life will be explored with questionnaires. The following questionnaires will be used to measure the quality of life: EORTC QLQ-C30, EORTC-QLQ-H&N35, EQ-5D-5L, and Shoulder Disability Questionnaire (SDQ) [15–18].

Cost-effectiveness analysis will be performed from a societal perspective with a time horizon of three months comparing usual care with a situation in which [^18^F]FDG PET/CT would be implemented before transoral excision and SLNB. Costs of different procedures will be calculated with a bottom-up calculation, including duration of the surgery in the electronic Case Report Forms (Castor EDC) [19]. Associated healthcare use after both the transoral excision and SLNB as well as the neck dissection, such as hospital admission days, will be collected from the electronic hospital records in every participating hospital and linked to Dutch unit costs. Both the Medical Consumption Questionnaire (iMCQ) and the Productivity Cost Questionnaire (iPCQ) will be sent to collect costs outside the hospital and productivity losses for each patient.

Furthermore, to gain insight into the patients’ preferences and experience with diagnostic modalities, semi-structured interviews will be conducted with patients at University Medical Center Utrecht until no new information, themes or insights emerge during data collection and data saturation is achieved.

### Sample size calculation

Since it is unlikely that [^18^F]FDG PET/CT will detect micrometastases with a sensitivity high enough to refrain from (elective) neck dissection if [^18^F]FDG PET/CT is negative, the focus of this study will be on a PPV high enough to avoid SLNB with second stage neck dissection if [^18^F]FDG PET/CT is positive. The sample size calculation is made on the number of early-stage OSCC patients, who undergo [^18^F]FDG PET/CT, needed to reduce reliably the number of (positive) SLNB by 25%.

No studies are available that use histopathological examination of neck dissection specimens or SLNB with follow-up as the reference standard while varying cut-off values of objective scoring parameters, such as SUVmax, to achieve a high positive predictive value (PPV) for the detection of occult lymph node metastases. The study of Peltenburg et al. compared SUVmax and lymph node size with the outcome of US-FNAC [20]. Although the sensitivity of US-FNAC is limited, its specificity is near 100%. In 52 patients with lymph node smaller than 10 mm (radiologically negative) SUVmax was > 4.9 in 17 patients of whom 12 had US-FNAC positive lymph nodes. Thus, if patients with SUVmax > 4.9 would undergo directly a neck dissection in at least 12 patients a SLNB (with neck dissection as a second stage operation if SLNB is positive) could correctly be avoided. Using this SUVmax cut-off value a reduction of SLNB of 32.7% can be obtained and only a few unnecessary neck dissection will be performed.

To show with an 80% power and a one-sided alpha of 0.05 that the reduction of SLNB of 0.327 is not inferior to 0.25, we need 143 patients. This sample size was calculated using the PASS power software, option: non-inferiority test for one proportion, exact test. As explained before it is expected that 4.2% of patients have to be excluded because of positive US-FNAC after to [^18^F]FDG PET/CT. Further drop-out rate for this study is expected to be 5%. After all, generally, patients want to have an extensive diagnostic work-up. [^18^F]FDG PET/CT is a routine diagnostic examination with a limited burden on the patient. Therefore, a total drop-out rate of (not more than) 10% is expected. Accordingly, this study’s population should consist of 159 patients to enable sufficient statistical power.

### Statistical analysis

The statistical analysis employs professional software (IBM SPSS Statistics) to present data as means with standard deviations for parametric continuous variables, and medians with interquartile ranges (IQR) for non-parametric variables. Normal distribution will be verified by using the Shapiro-Wilk test (p < 0.05 considered significant). Missing data are addressed via multiple imputation strategies. Results across multiple imputation data sets will be combined using Rubin’s rules. LASSO-based logistic regression analysis will be used to identify the optimal panel for distinguishing patients with metastases from those without.

For the primary endpoint, the reduction in (positive) SLNB and unnecessary neck dissections, including the associated false positives, will be calculated based on newly developed scoring criteria for [^18^F]FDG PET/CT. Models combining [^18^F]FDG PET/CT parameters will be analyzed for accuracy, assessing various cut-off values to determine optimal results, prioritizing high PPV to reduce SLNBs while maintaining acceptable sensitivity. Different scoring results (visual, semi-quantitative) and morphology characteristics of the [^18^F]FDG-positive lymph nodes will be compared between those with and without metastases using SLNB and/or 12-month follow-up as reference standards. Independent samples T-tests will be used for parametric data, and Friedman’s 2-way ANOVA and Wilcoxon signed rank tests for non-parametric data. Categorical data will be compared using χ²-tests and expressed as differences in proportions.

## Discussion

Currently, [^18^F]FDG PET/CT is not routinely used in the diagnostic work-up of early-stage OSCC [12]. Although promising results of the [^18^F]FDG PET/CT are reported, Stokkel et. al. and Yamazaiki et. al. showed that [^18^F]FDG PET/CT detects lymph node metastases in head and neck cancer more accurately than MRI and CT –two currently used diagnostic techniques to stage the neck in early-staged OSCC–, the evidence is mixed [2,6,21,22]. Comparison between studies are challenging due to variations in scan protocols, definitions of the N0 neck, criteria for PET positivity, scan reading methods, and reference standards [2,6].

To the best of our knowledge, the most recent systematic review and meta-analysis, which reviewed 18 studies (1044 patients), showed a pooled sensitivity for [^18^F]FDG PET/CT of 58%, and a specificity of 87%, PPV of 62%, and a NPV of 83% for cN0 head and neck cancer patients [2]. Later in 2022, Piotrowicz et al., showed a sensitivity of 68.8%, specificity of 85.1%, PPV of 59.9% and a NVP of 90% for [^18^F]FDG PET/CT in head and neck cancer patients [23]. Since PET imaging has improved considerably due to technical advances enabling integration of PET and CT devices, improvements in detector capabilities yielding higher image resolution, and optimization of head and neck acquisition parameters, new prospective studies are warranted [9].

This prospective multicenter cohort study is designed to address the gaps in the current literature regarding the use of [^18^F]FDG PET/CT in patients with cN0 OSCC. First of all, the most important distinction of the PETN0 study from current literature is our focus on developing scoring criteria with a high PPV to reduce the amount of SLNBs, whereas current literature is focused on sensitivity and NVP [2,23]. Focusing on achieving a high PPV allows us to select individuals with a high risk of cervical lymph node metastases, for whom neck dissection can be performed without necessitating an SLNB. Patients presenting a negative [^18^F]FDG PET/CT will still undergo SLNB, thereby ensuring that occult lymph node metastases, which might not be detected by [^18^F]FDG PET/CT, are not missed. This method for implementing [^18^F]FDG PET/CT in early-stage OSCC had not been previously described in literature and potentially offers a strong strategy to reduce the amount of SLNBs, improve patient treatment outcomes and prevent the oversight of (occult) metastases in patients with early-stage OSCC.

Another limitation in the existing literature is the reference standard that has been used. Currently, the diagnostic accuracy of [^18^F]FDG PET/CT is determined based on histopathological outcomes from neck dissections [2,23]. Routine histopathological examination of neck dissection specimens can miss micrometastases, whereas step serial sectioning and immunohistochemistry, as performed in SLNB, can increase detection rates by as much as 15.2% [24]. Thus, histopathological results of SLNB and follow-up in case of a negative SLNB, is the most reliable reference standard [25]. Our prospective study uses SLNB and follow-up in case of a negative SLNB as reference standard. By employing this method, we aim to achieve a more reliable assessment of the diagnostic accuracy of [^18^F]FDG PET/CT compared to the current literature [2].

To minimize the likelihood of lymph node metastasis occurring between the [^18^F]FDG PET/CT scan and SLNB, this study aims to perform both diagnostic tests within one week, and no later than three weeks apart. This timeline falls within the established Dutch national guidelines for OSCC. No complications are expected for the [^18^F]FDG PET/CT and contrast-enhanced CT of the neck.

In short, [^18^F]FDG PET/CT is not routinely used in clinical staging of the neck for early-stage OSCC, but several promising results for the detection of occult LNM have been reported. Accurate staging of the clinical positive neck using non-invasive screening methods is beneficial, as a subsequent completed neck dissection following a positive SLNB presents logistics challenges and an increased risk of complications. By developing scoring criteria within the PETN0 study for the [^18^F]FDG PET/CT with a high PPV, it becomes possible to identify a subset of patients high at risk for cervical lymph node metastases, who can undergo an neck dissection without the need for SLNB. In case of a negative [^18^F]FDG PET/CT, SLNB will still be performed. This approach ensures that occult metastases missed by [^18^F]FDG PET/CT are still detected through SLNB, while reducing the need for SLNB procedures overall, thereby optimizing treatment outcomes while minimizing unnecessary interventions.

## Conclusion

Patients (n = 159), diagnosed with early-stage OSCC (T1-3N0M0, only when T3 is assessed based on tumor dimensions of >2 cm and ≤4 cm with DOI > 10 mm), and candidate for transoral excision and SLNB, are included in the PETN0 study. The aim of this study is to develop scorings criteria for [^18^F]FDG PET/CT with a high PPV to reduce the need for SLNB in patients with cN0 OSCC. The study procedure exists of an [^18^F]FDG PET/CT, a contrast-enhanced CT of the neck and questionnaires to evaluate the quality of life and cost effectiveness. In addition, we anticipate to provide a more reliable assessment of the diagnostic accuracy of [^18^F]FDG PET-CT in patients with early-stage OSCC by utilizing a more accurate reference standard compared to existing literature. Because it is not expected that [^18^F]FDG PET/CT detect all (occult) lymph node metastases, SLNB will be necessary in cases of a negative [^18^F]FDG PET/CT.

## Supporting information

S1 Spirit Checklist. (DOCX)

S2 SPIRIT-Figure PETN0-study protocol one. (TIF)

S3 C1. Onderzoeksprotocol 1.2. (PDF)
