# Supplementary material for: [18F]FDG PET/CT to reduce the need for sentinel lymph node biopsy in early-stage oral cancer: PETN0-study protocol
Source: PLoS One. 2025 Jul 1;20(7):e0325032. doi: 10.1371/journal.pone.0325032 (PMC12212575; doi:10.1371/journal.pone.0325032)
Supplement: S1 — (DOCX) [file pone.0325032.s001.docx]

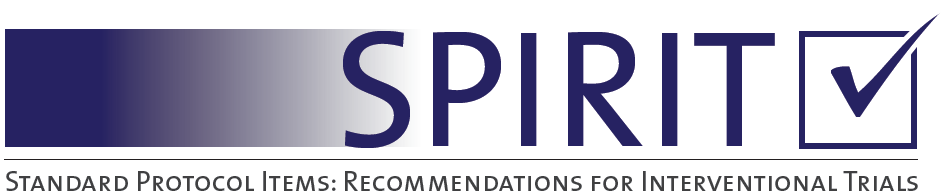


SPIRIT 2013 Checklist: Recommended items to address in a clinical trial protocol and related documents*

| Section/item | ItemNo | Description |
| --- | --- | --- |
| **Administrative information** | | |
| Title | 1 | Descriptive title identifying the study design, population, interventions, and, if applicable, trial acronym  Reference: Manuscript lines 1-2, Protocol Title page  "FDG-PET/CT to reduce the need for sentinel lymph node biopsy in early-stage oral cancer: PETN0-study protocol". |
| Trial registration | 2a | Trial identifier and registry name. If not yet registered, name of intended registry Reference: Manuscript lines 110-112, Protocol footnotes.  Registered under NL83442.041.22. |
|  | 2b | All items from the World Health Organization Trial Registration Data  Reference: Protocol, section on trial registration.  Includes all required elements; found in study protocol. |
| Protocol version | 3 | Date and version identifier Reference: See file ‘C1. Research protocol NL83442.041.22 version 1.3 dd. 15-08-2024_clean_signed’ date and version identifier are found in footnote. |
| Funding | 4 | Sources and types of financial, material, and other support Reference: Manuscript lines 109-110.  Funded by KWF Dutch Cancer Society, project number 14109. |
| Roles and responsibilities | 5a | Names, affiliations, and roles of protocol contributors Reference: manuscript lines 7-42.: |
|  | 5b | Name and contact information for the trial sponsor Reference: manuscript lines 45-46.  Sponsor: University Medical Center Utrecht (UMC Utrecht), contact sponsor: prof. dr. R. de Bree. |
|  | 5c | Role of study sponsor and funders, if any, in study design; collection, management, analysis, and interpretation of data; writing of the report; and the decision to submit the report for publication, including whether they will have ultimate authority over any of these activities Reference: manuscript lines 334-345 and file ‘C1. Research protocol NL83442.041.22 version 1.3 dd. 15-08-2024_clean_signed’. |
|  | 5d | Composition, roles, and responsibilities of the coordinating centre, steering committee, endpoint adjudication committee, data management team, and other individuals or groups overseeing the trial, if applicable (see Item 21a for data monitoring committee) Reference: manuscript lines 334-345 and file ‘C1. Research protocol NL83442.041.22 version 1.3 dd. 15-08-2024_clean_signed’. |
| Introduction |  |  |
| Background and rationale | 6a | Description of research question and justification for undertaking the trial, including summary of relevant studies (published and unpublished) examining benefits and harms for each intervention Reference: Manuscript lines 73-106 and file ‘C1. Research protocol NL83442.041.22 version 1.3 dd. 15-08-2024_clean_signed’.  Discusses importance of early detection of lymph node metastases and role of FDG-PET/CT. |
|  | 6b | Explanation for choice of comparators Reference: manuscript lines 73-106 and file ‘C1. Research protocol NL83442.041.22 version 1.3 dd. 15-08-2024_clean_signed’. Justification for comparing FDG-PET/CT with SLNB. |
| Objectives | 7 | Specific objectives or hypotheses Reference: Manuscript lines 99-106 and file ‘C1. Research protocol NL83442.041.22 version 1.3 dd. 15-08-2024_clean_signed’.  Primary objective: Reduce need for SLNB.  Secondary objectives: Optimize PET/CT scoring criteria, assess accuracy, compare QoL, and costs. |
| Trial design | 8 | Description of trial design including type of trial (eg, parallel group, crossover, factorial, single group), allocation ratio, and framework (eg, superiority, equivalence, noninferiority, exploratory) Reference: manuscript line 109 and file ‘C1. Research protocol NL83442.041.22 version 1.3 dd. 15-08-2024_clean_signed’.  Prospective multicenter cohort study. |
| Methods: Participants, interventions, and outcomes | | |
| Study setting | 9 | Description of study settings (eg, community clinic, academic hospital) and list of countries where data will be collected. Reference to where list of study sites can be obtained Reference: manuscript lines 109-116 and file ‘C1. Research protocol NL83442.041.22 version 1.3 dd. 15-08-2024_clean_signed’.  Conducted in eight head and neck centers of the Dutch Head and Neck Society: University Medical Center Utrecht, Amsterdam University Medical Center location Vrije Universiteit Amsterdam, Haaglanden Medical Center, Leiden University Medical Center, Maastricht University Medical Center+, The Netherlands Cancer Institute-Antoni van Leeuwenhoek, Radboud University Medical Center and University Medical Center Groningen. |
| Eligibility criteria | 10 | Inclusion and exclusion criteria for participants. If applicable, eligibility criteria for study centres and individuals who will perform the interventions (eg, surgeons, psychotherapists) Reference: manuscript lines 117-124 and and file ‘C1. Research protocol NL83442.041.22 version 1.3 dd. 15-08-2024_clean_signed’ protocol section 4.2. |
| Interventions | 11a | Interventions for each group with sufficient detail to allow replication, including how and when they will be administered Reference: Protocol section 5.1 and manuscript lines 126-139.  FDG-PET/CT performed within 3 weeks before SLNB. |
|  | 11b | Criteria for discontinuing or modifying allocated interventions for a given trial participant (eg, drug dose change in response to harms, participant request, or improving/worsening disease) Reference: Manuscript lines 141-151 and file ‘C1. Research protocol NL83442.041.22 version 1.3 dd. 15-08-2024_clean_signed’. |
|  | 11c | Strategies to improve adherence to intervention protocols, and any procedures for monitoring adherence (eg, drug tablet return, laboratory tests) Reference: Manuscript lines 141-143, 159, 170-171 and file ‘C1. Research protocol NL83442.041.22 version 1.3 dd. 15-08-2024_clean_signed’ |
|  | 11d | Relevant concomitant care and interventions that are permitted or prohibited during the trial Reference: Manuscript lines 146-149 and file ‘C1. Research protocol NL83442.041.22 version 1.3 dd. 15-08-2024_clean_signed’. |
| Outcomes | 12 | Primary, secondary, and other outcomes, including the specific measurement variable (eg, systolic blood pressure), analysis metric (eg, change from baseline, final value, time to event), method of aggregation (eg, median, proportion), and time point for each outcome. Explanation of the clinical relevance of chosen efficacy and harm outcomes is strongly recommended Reference: manuscript lines 177-182 (Table 3) and file ‘C1. Research protocol NL83442.041.22 version 1.3 dd. 15-08-2024_clean_signed’. |
| Participant timeline | 13 | Time schedule of enrolment, interventions (including any run-ins and washouts), assessments, and visits for participants. A schematic diagram is highly recommended (see Figure) Reference: manuscript lines 127-138 (Fig 1; SPIRIT schedule) and file ‘C1. Research protocol NL83442.041.22 version 1.3 dd. 15-08-2024_clean_signed’. |
| Sample size | 14 | Estimated number of participants needed to achieve study objectives and how it was determined, including clinical and statistical assumptions supporting any sample size calculations Reference: manuscript lines 119, and 217-264 and file ‘C1. Research protocol NL83442.041.22 version 1.3 dd. 15-08-2024_clean_signed’. 159 participants planned. |
| Recruitment | 15 | Strategies for achieving adequate participant enrolment to reach target sample size Reference: manuscript lines 217-224 and file ‘C1. Research protocol NL83442.041.22 version 1.3 dd. 15-08-2024_clean_signed’. Multicenter recruitment strategy. |
| **Methods: Assignment of interventions (for controlled trials)** | | |
| Allocation: |  |  |
| Sequence generation | 16a | Method of generating the allocation sequence (eg, computer-generated random numbers), and list of any factors for stratification. To reduce predictability of a random sequence, details of any planned restriction (eg, blocking) should be provided in a separate document that is unavailable to those who enrol participants or assign interventions Not applicable (NA) |
| Allocation concealment mechanism | 16b | Mechanism of implementing the allocation sequence (eg, central telephone; sequentially numbered, opaque, sealed envelopes), describing any steps to conceal the sequence until interventions are assigned NA |
| Implementation | 16c | Who will generate the allocation sequence, who will enrol participants, and who will assign participants to interventions NA |
| Blinding (masking) | 17a | Who will be blinded after assignment to interventions (eg, trial participants, care providers, outcome assessors, data analysts), and how Reference: Manuscript lines 162-164 and file ‘C1. Research protocol NL83442.041.22 version 1.3 dd. 15-08-2024_clean_signed’. FDG-PET/CT results of the neck will be blinded for sentinel lymph node biopsy. |
|  | 17b | If blinded, circumstances under which unblinding is permissible, and procedure for revealing a participant’s allocated intervention during the trial Reference: Manuscript lines 166-170 and file ‘C1. Research protocol NL83442.041.22 version 1.3 dd. 15-08-2024_clean_signed’. |
| **Methods: Data collection, management, and analysis** | | |
| Data collection methods | 18a | Plans for assessment and collection of outcome, baseline, and other trial data, including any related processes to promote data quality (eg, duplicate measurements, training of assessors) and a description of study instruments (eg, questionnaires, laboratory tests) along with their reliability and validity, if known. Reference to where data collection forms can be found, if not in the protocol Reference: file ‘C1. Research protocol NL83442.041.22 version 1.3 dd. 15-08-2024_clean_signed’ |
|  | 18b | Plans to promote participant retention and complete follow-up, including list of any outcome data to be collected for participants who discontinue or deviate from intervention protocols Reference: file ‘C1. Research protocol NL83442.041.22 version 1.3 dd. 15-08-2024_clean_signed’ Planned follow-up of 12 months and 24 months in the long-term study. |
| Data management | 19 | Plans for data entry, coding, security, and storage, including any related processes to promote data quality (eg, double data entry; range checks for data values). Reference to where details of data management procedures can be found, if not in the protocol Reference: file ‘C1. Research protocol NL83442.041.22 version 1.3 dd. 15-08-2024_clean_signed’ Electronic database Is used (Castor EDC). |
| Statistical methods | 20a | Statistical methods for analysing primary and secondary outcomes. Reference to where other details of the statistical analysis plan can be found, if not in the protocol Reference: Manuscript lines 246-264 and file ‘C1. Research protocol NL83442.041.22 version 1.3 dd. 15-08-2024_clean_signed’. |
|  | 20b | Methods for any additional analyses (eg, subgroup and adjusted analyses) Reference: manuscript lines 246-264 and file ‘C1. Research protocol NL83442.041.22 version 1.3 dd. 15-08-2024_clean_signed’. |
|  | 20c | Definition of analysis population relating to protocol non-adherence (eg, as randomised analysis), and any statistical methods to handle missing data (eg, multiple imputation) Reference: Manuscript lines 246-264 (250) and file ‘C1. Research protocol NL83442.041.22 version 1.3 dd. 15-08-2024_clean_signed’. |
| **Methods: Monitoring** | | |
| Data monitoring | 21a | Composition of data monitoring committee (DMC); summary of its role and reporting structure; statement of whether it is independent from the sponsor and competing interests; and reference to where further details about its charter can be found, if not in the protocol. Alternatively, an explanation of why a DMC is not needed Reference: file ‘C1. Research protocol NL83442.041.22 version 1.3 dd. 15-08-2024_clean_signed’ |
|  | 21b | Description of any interim analyses and stopping guidelines, including who will have access to these interim results and make the final decision to terminate the trial NA, no interim analysis will be conducted. |
| Harms | 22 | Plans for collecting, assessing, reporting, and managing solicited and spontaneously reported adverse events and other unintended effects of trial interventions or trial conduct Reference: file ‘C1. Research protocol NL83442.041.22 version 1.3 dd. 15-08-2024_clean_signed’ |
| Auditing | 23 | Frequency and procedures for auditing trial conduct, if any, and whether the process will be independent from investigators and the sponsor  Reference: file ‘C1. Research protocol NL83442.041.22 version 1.3 dd. 15-08-2024_clean_signed’ |
| Ethics and dissemination | | |
| Research ethics approval | 24 | Plans for seeking research ethics committee/institutional review board (REC/IRB) approval  Reference: Manuscript line 109 and file ‘C1. Research protocol NL83442.041.22 version 1.3 dd. 15-08-2024_clean_signed’.  The METC (Medical Ethics Review Committee) approval has already been obtained prior to the commencement of the study. The necessary documentation, including the study protocol and informed consent forms, was submitted for review, and all feedback from the committee was addressed to ensure compliance with ethical standards. |
| Protocol amendments | 25 | Plans for communicating important protocol modifications (eg, changes to eligibility criteria, outcomes, analyses) to relevant parties (eg, investigators, REC/IRBs, trial participants, trial registries, journals, regulators) Reference: file ‘C1. Research protocol NL83442.041.22 version 1.3 dd. 15-08-2024_clean_signed’ Any changes (amendments) will be communicated to the METC. |
| Consent or assent | 26a | Who will obtain informed consent or assent from potential trial participants or authorised surrogates, and how (see Item 32) See file ‘C1. Research protocol NL83442.041.22 version 1.3 dd. 15-08-2024_clean_signed’ |
|  | 26b | Additional consent provisions for collection and use of participant data and biological specimens in ancillary studies, if applicable Consent for this is included in the informed consent signed by participants. |
| Confidentiality | 27 | How personal information about potential and enrolled participants will be collected, shared, and maintained in order to protect confidentiality before, during, and after the trial Reference: file ‘C1. Research protocol NL83442.041.22 version 1.3 dd. 15-08-2024_clean_signed’. |
| Declaration of interests | 28 | Financial and other competing interests for principal investigators for the overall trial and each study site Reference: Manuscript submission form.  No conflicts of interest declared. |
| Access to data | 29 | Statement of who will have access to the final trial dataset, and disclosure of contractual agreements that limit such access for investigators Reference: file ‘C1. Research protocol NL83442.041.22 version 1.3 dd. 15-08-2024_clean_signed’ |
| Ancillary and post-trial care | 30 | Provisions, if any, for ancillary and post-trial care, and for compensation to those who suffer harm from trial participation NA |
| Dissemination policy | 31a | Plans for investigators and sponsor to communicate trial results to participants, healthcare professionals, the public, and other relevant groups (eg, via publication, reporting in results databases, or other data sharing arrangements), including any publication restrictions Reference: file ‘C1. Research protocol NL83442.041.22 version 1.3 dd. 15-08-2024_clean_signed’ |
|  | 31b | Authorship eligibility guidelines and any intended use of professional writers Reference: file ‘C1. Research protocol NL83442.041.22 version 1.3 dd. 15-08-2024_clean_signed’ |
|  | 31c | Plans, if any, for granting public access to the full protocol, participant-level dataset, and statistical code Reference: file ‘C1. Research protocol NL83442.041.22 version 1.3 dd. 15-08-2024_clean_signed’ |
| Appendices |  |  |
| Informed consent materials | 32 | Model consent form and other related documentation given to participants and authorised surrogates Reference: file ‘C1. Research protocol NL83442.041.22 version 1.3 dd. 15-08-2024_clean_signed’ and ‘DEFINITIEF NA METC GOEDKEURING E1E2. Proefpersoneninformatie NL83442.041.22 versie 1.2 dd. 14-06-2024_Clean’. |
| Biological specimens | 33 | Plans for collection, laboratory evaluation, and storage of biological specimens for genetic or molecular analysis in the current trial and for future use in ancillary studies, if applicable NA |

*It is strongly recommended that this checklist be read in conjunction with the SPIRIT 2013 Explanation & Elaboration for important clarification on the items. Amendments to the protocol should be tracked and dated. The SPIRIT checklist is copyrighted by the SPIRIT Group under the Creative Commons “[Attribution-NonCommercial-NoDerivs 3.0 Unported](http://www.creativecommons.org/licenses/by-nc-nd/3.0/)” license.
