# Supplementary figures and images for: [18F]FDG PET/CT to reduce the need for sentinel lymph node biopsy in early-stage oral cancer: PETN0-study protocol
Source: PLoS One. 2025 Jul 1;20(7):e0325032. doi: 10.1371/journal.pone.0325032 (PMC12212575; doi:10.1371/journal.pone.0325032)

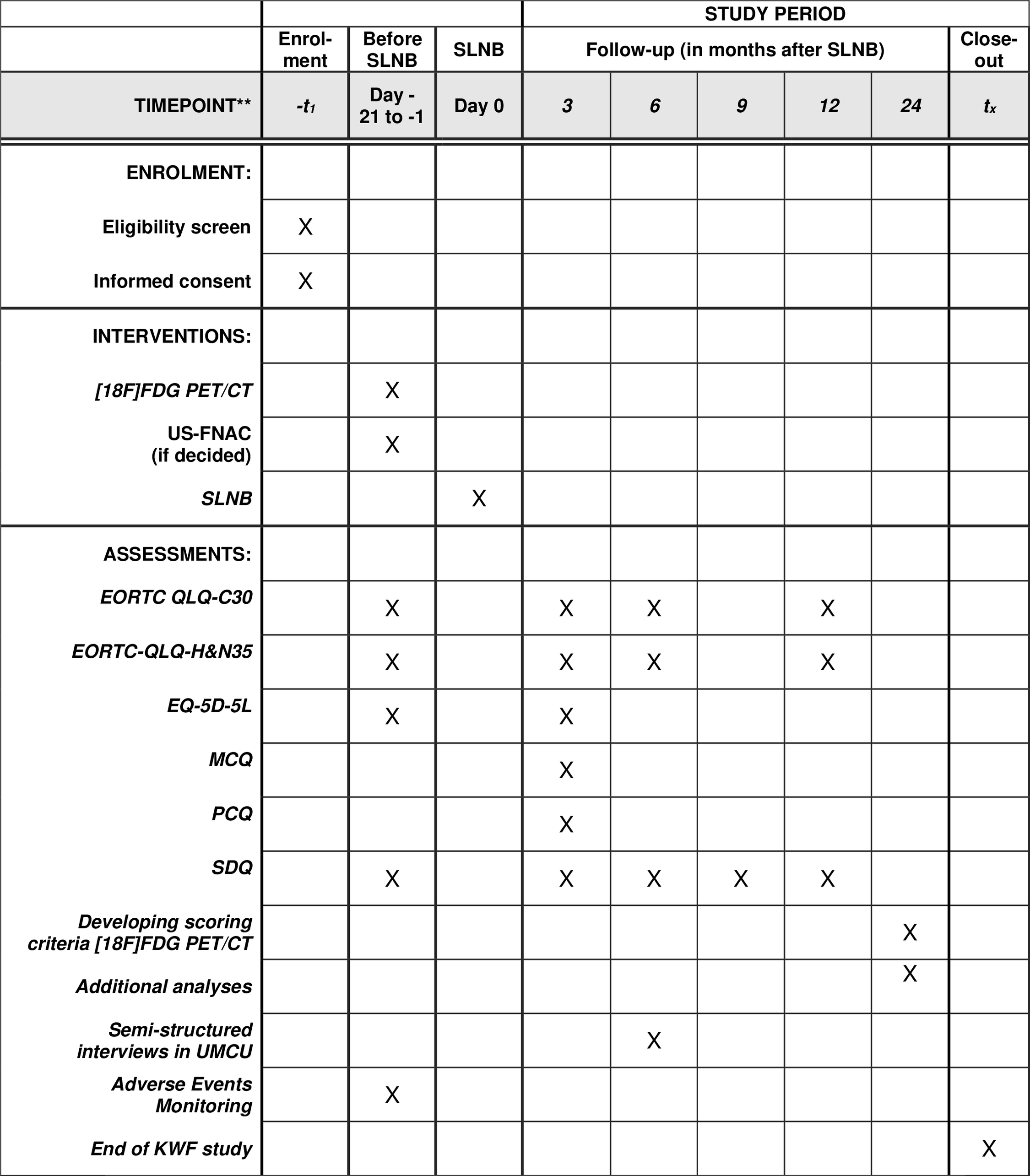

Supplement: S2 — (TIF) [file pone.0325032.s002.tif]
