# Supplementary material for: [18F]FDG PET/CT to reduce the need for sentinel lymph node biopsy in early-stage oral cancer: PETN0-study protocol
Source: PLoS One. 2025 Jul 1;20(7):e0325032. doi: 10.1371/journal.pone.0325032 (PMC12212575; doi:10.1371/journal.pone.0325032)
Supplement: S3 — (PDF) [file pone.0325032.s003.pdf]

FDG-PET/CT to reduce the need for sentinel lymph node biopsy in early-stage oral cancer

PROTOCOL TITLE '‘FDG-PET/CT to reduce the need for sentinel lymph node biopsy in early-stage oral cancer’

**Protocol ID**	NL83442.041.22
**Short title**	PETN0
**EudraCT number**	Not applicable
**Version**	1.2
**Date**	12-09-2023-2023
**Coordinating investigator/project leader**	Prof. dr. R. de Bree<br>Department Head and Neck Surgical Oncology<br>Division Imaging & Oncology<br>University Medical Center Utrecht<br>Heidelberglaan 100<br>3584 CX Utrecht, the Netherlands<br>Tel +31 88 75 562 65<br>e-mail: R.deBree@umcutrecht.nl;
**Principal investigator(s) (in Dutch: hoofdonderzoeker/ uitvoerder)**<br><br>**Multicenter research: per site**	Prof. dr. R. de Bree<br>Department Head and Neck Surgical Oncology<br>UMC Utrecht, Division Imaging & Oncology<br>e-mail: R.deBree@umcutrecht.nl<br><br>Prof. dr. R.P. Takes<br>Department Otolaryngology – Head and Neck Surgical Oncology<br>Radboud University Medical Center<br>Robert.Takes@radboudumc.nl;<br><br>Dr. S.A.H.J. de Visscher<br>Department Maxillofacial Surgery<br>University Medical Center Groningen<br>s.a.h.j.de.visscher@umcg.nl;<br><br>Prof. dr. C.R. Leemans<br>Department Otolaryngology – Head and Neck Surgical Oncology<br>Amsterdam University Medical Center, location VUmc

	cr.leemans@amsterdamumc.nl<br><br>Drs. R-J.E. Sedee<br>Department Otolaryngology<br>Haaglanden Medical Center<br>r.sedee@haaglandenmc.nl<br><br>Dr. M. Lacko<br>Department Otolaryngology - Head and Neck Surgical Oncology<br>Maastricht University Medical Center+<br>Martin.lacko@mumc.nl<br><br>Dr. S.L. van Egmond<br>Department Head and Neck Surgical Oncology<br>Leids University Medical Center<br>S.L.van_Egmond@lumc.nl<br><br>Dr. M. Klop<br>Department Head and Neck Surgery and Oncology<br>Netherlands Cancer Institute<br>m.klop@nki.nl
**Subinvestigators UMC Utrecht**	Dr. B. de Keizer<br>UMC Utrecht, Division Imaging & Oncology<br>e-mail: B.deKeizer@umcutrecht.nl<br><br>Drs. R.S. Tellman<br>UMC Utrecht, Division Imaging & Oncology<br>e-mail: mailto:r.s.tellman@umcutrecht.nl
**Sponsor (in Dutch: verrichter/opdrachtgever)**	University Medical Center Utrecht (UMC Utrecht)
**Subsidising party**	KWF 14304
**Independent expert (s)**	Dr. P.A.H. Doornaert<br>Department of Radiation Oncology<br>University Medical Center Utrecht

**Laboratory sites**	Not applicable
**Pharmacy <*if applicable*>**	Not applicable

PROTOCOL SIGNATURE SHEET

Name	Signature	Date
**manager research**<br>Prof. dr. H.M. Verkooijen<br>Manager Research<br>University Medical Center Utrecht, Division Imaging & Oncology		12-09-2023
**Coordinating Investigator/Project leader/Principal Investigator:**<br>Prof. dr. R. de Bree<br>Otolaryngologist / Head and Neck Surgeon<br>University Medical Center Utrecht, Division Imaging & Oncology		12-09-2023

TABLE OF CONTENTS

1. STUDY DESIGN ....
2. STUDY POPULATION 19
2.1 Population (base) ....
2.2 Inclusion criteria 19
2.3 Exclusion criteria 19
2.4 Sample size calculation ....
3. TREATMENT OF SUBJECTS 21
3.1 Investigational product/treatment...21
3.2 Use of co-intervention (if applicable) 21
3.3 Escape medication (if applicable) 21
4. INVESTIGATIONAL PRODUCT 22
4.1 Name and description of investigational product(s) 22
4.2 Summary of findings from non-clinical studies 22
4.3 Summary of findings from clinical studies 22
4.4 Summary of known and potential risks and benefits 22
4.5 Description and justification of route of administration and dosage.........................22
4.6 Dosages, dosage modifications and method of administration 22
4.7 Preparation and labelling of Investigational Medicinal Product 22
4.8 Drug accountability 22
5. NON-INVESTIGATIONAL PRODUCT 23
5.1 Name and description of non-investigational product(s) 23
5.2 Summary of findings from non-clinical studies 23
5.3 Summary of findings from clinical studies 23
5.4 Summary of known and potential risks and benefits 23
5.5 Description and justification of route of administration and dosage.........................23
5.6 Dosages, dosage modifications and method of administration 23
5.7 Preparation and labelling of Non Investigational Medicinal Product........................23
5.8 Drug accountability 23
6. METHODS 24
6.1 Study parameters/endpoints..24
6.1.1 Main study parameter/endpoint 24
6.1.2 Secondary study parameters/endpoints (if applicable) 24
6.1.3 Other study parameters (if applicable)...25
6.2 Randomisation, blinding and treatment allocation 26
6.3 Study procedures 26

6.4 Withdrawal of individual subjects ... 26
6.4.1 Specific criteria for withdrawal (if applicable) ... 26
6.5 Replacement of individual subjects after withdrawal**Fout! Bladwijzer niet gedefinieerd.**
6.6 Follow-up of subjects withdrawn from treatment ... 26
6.7 Premature termination of the study ... 26
7. SAFETY REPORTING ... 27
7.1 Temporary halt for reasons of subject safety ... 27
7.2 AEs, SAEs and SUSARs ... 27
7.2.1 Adverse events (AEs) ... 27
7.2.2 Serious adverse events (SAEs) ... 27
7.2.3 Suspected unexpected serious adverse reactions (SUSARs) ... 28
7.3 Annual safety report ... 28
7.4 Follow-up of adverse events ... 28
7.5 [Data Safety Monitoring Board (DSMB) / Safety Committee] ... 28
8. STATISTICAL ANALYSIS ... 29
8.1 Primary study parameter(s) ... 29
8.2 Secondary study parameter(s) ... 29
8.3 Other study parameters ... 32
8.4 Interim analysis (if applicable) ... 33
9. ETHICAL CONSIDERATIONS ... 34
9.1 Regulation statement ... 34
9.2 Recruitment and consent ... 34
9.3 Objection by minors or incapacitated subjects (if applicable) ... 35
9.4 Benefits and risks assessment, group relatedness ... 35
9.5 Compensation for injury ... 35
9.6 Incentives (if applicable) ... 35
10. ADMINISTRATIVE ASPECTS, MONITORING AND PUBLICATION ... 36
10.1 Handling and storage of data and documents ... 36
10.2 Monitoring and Quality Assurance ... 36
10.3 Amendments ... 36
10.4 Annual progress report ... 36
10.5 Temporary halt and (prematurely) end of study report ... 37
10.6 Public disclosure and publication policy ... 37
11. STRUCTURED RISK ANALYSIS ... 38
11.1 Potential issues of concern ... 38
11.2 Synthesis ... 38

12. REFERENCES 39

LIST OF ABBREVIATIONS AND RELEVANT DEFINITIONS

ABR	General Assessment and Registration form (ABR form), the application form that is required for submission to the accredited Ethics Committee; in Dutch: Algemeen Beoordelings- en Registratieformulier (ABR-formulier)
AE	Adverse Event
AR	Adverse Reaction
CA	Competent Authority
CCMO	Central Committee on Research Involving Human Subjects; in Dutch: Centrale Commissie Mensgebonden Onderzoek
CND	Completed Neck Dissection
CT	Computed Tomography
CV	Curriculum Vitae
DSMB	Data Safety Monitoring Board
END	Elective Neck Dissection
EU	European Union
EudraCT	European drug regulatory affairs Clinical Trials
FDG	FluoroDeoxyGlucose
GCP	Good Clinical Practice
GDPR	General Data Protection Regulation; in Dutch: Algemene Verordening Gegevensbescherming (AVG)
HNSCC	Head and Neck Squamous Cell Carcinoma
IB	Investigator’s Brochure
IC	Informed Consent
ICG	Indocyanine Green
IMP	Investigational Medicinal Product
IMPD	Investigational Medicinal Product Dossier
LNM	Lymph Node Metastases
MCQ	Medical Consumption Questionnaire
METC	Medical research ethics committee (MREC); in Dutch: medisch-ethische toetsingscommissie (METC)
NCCN	National Comprehensive Cancer Network
NFU	Nederlandse Federatie van Universitair Medische Centra
NICE	National Institute for Health and Care Excellence
KWF	Koningin Wilhelmina Fonds voor de Nederlandse Kankerbestrijding
OSCC	Oral Squamous Cell Carcinoma
PCQ	Productivity Cost Questionnaire

**PET**	**Positron Emission Tomography**
**(S)AE**	**(Serious) Adverse Event**
**SDQ**	**Shoulder Disability Questionnaire**
**SLN**	**Sentinel Lymph Node**
**SLNB**	**Sentinel Lymph Node Biopsy**
**SPC**	**Summary of Product Characteristics; in Dutch: officiële productinformatie IB1-tekst**
**Sponsor**	**The sponsor is the party that commissions the organisation or performance of the research, for example a pharmaceutical company, academic hospital, scientific organisation or investigator. A party that provides funding for a study but does not commission it is not regarded as the sponsor, but referred to as a subsidising party.**
**SUSAR**	**Suspected Unexpected Serious Adverse Reaction**
**UAVG**	**Dutch Act on Implementation of the General Data Protection Regulation; in Dutch: Uitvoeringswet AVG**
**USgFNAC**	**Ultrasound Guided Fine Needle Aspiration Cytology**
**WMO**	**Medical Research Involving Human Subjects Act; in Dutch: Wet Medisch-wetenschappelijk Onderzoek met Mensen**

SUMMARY

Rationale: In head and neck squamous cell carcinoma (HNSCC) the detection of lymph node metastases (LNM) is highly important because adequate treatment of the neck, i.e. neck dissection and/or radiotherapy is available. Sentinel lymph node biopsy (SLNB) can reliably detect occult LNM in early-stage (cT1-2N0) oral squamous cell carcinoma (OSCC) and is now incorporated in many national guidelines. Management of the neck based on SLNB has some limitations. SLNB remains an invasive surgical procedure with associated morbidity. An eventual subsequent complete neck dissection (CND) is a second stage procedure in 30% of patients and should proceed in a timely fashion, which may be a logistic problem. Moreover, subsequent CND is more challenging than elective neck dissection (END) and harbours a higher risk of complications. Although promising results have been reported, the role of FDG-PET/CT to detect occult LNM in HNSCC patients is unclear. Routine histopathological examination of neck dissection specimens can miss micrometastases, whereas step serial sectioning and immunohistochemistry (as performed in SLNB) can increase the yield by as much as 15.2%. Therefore, the most reliable reference standard, which has not been used in PET/CT studies before, is SLNB and follow-up without treatment of the neck in case of negative SLNB. PET imaging has improved considerably due to technical advances, improvements in detector capabilities yielding higher image resolution, and optimization of head and neck acquisition parameters. The number of SLNB procedures and second stage CND can be reduced by performing direct neck dissection without SLNB procedures in patients in whom occult LNM can be predicted by FDG-PET/CT with very high positive predictive value (PPV). By varying cut-off levels scoring criteria can be developed and optimized to predict the presence of lymph node metastases with high PPV instead of high sensitivity, which is usually done. When focused on high PPV the sensitivity will probably be lower, but missed LNM will then be detected by SLNB when performed after negative FDG-PET/CT.

Objective: In this study, we aim to investigate the possibility of FDG-PET/CT to reduce the number of (positive) SLNB procedures in patients with early stage oral squamous cell carcinoma and a clinically negative neck.

Study design: This study is designed as a prospective Dutch multicentre cohort study.

Study population: A total of 159 patients with newly diagnosed early-stage OSCC (cT1-3, N0, M0) scheduled for transoral resection and SLNB, will be included in a multicentre study.

Intervention: One FDG-PET/CT before treatment and questionnaires (before treatment and at 3, 6, 9, and 12 months after treatment).

Main study parameters/endpoints: The primary endpoint is the reduction of (positive) SLNB procedures. Secondary endpoints are PPV, sensitivity, specificity, negative predictive

value and accuracy of FDG-PET/CT for the detection of cervical lymph node metastasis in cN0 OSCC patients using different scoring criteria.

Nature and extent of the burden and risks associated with participation, benefit, and group relatedness: Patients will undergo additional FDG-PET/CT before treatment. The SNLB procedure is standard care for these patients in the participating centers. Early-stage OSCC patients usually do not undergo FDG-PET/CT. The results of the FDG-PET/CT of the neck will not be utilized for the treatment of the neck. However, the FDG-PET/CT may detect other tumor sites in the thorax or upper abdomen. Patients may benefit from this, but false-positive findings may give a burden on the patients. This may include other additional examinations to confirm PET/CT findings. FDG-PET/CT is considered a safe procedure, with limited radiation exposure. Therefore, we conclude the risk is negligible for this study according to the NFU guidelines.

1. INTRODUCTION AND RATIONALE

In the Netherlands 3017 patients were diagnosed with head and neck squamous cell carcinoma (HNSCC) in 2020, of whom 920 had oral squamous cell carcinoma (OSCC). In HNSCC the detection of lymph node metastases (LNM) is highly important because adequate treatment of the neck, i.e. neck dissection and/or radiotherapy is available. Sentinel lymph node biopsy (SLNB) can reliably detect occult LNM in early-stage (cT1-2N0) OSCC and is now incorporated in many national guidelines including those from The Netherlands, United Kingdom (NICE), and United States of America (NCCN) (1-3). Management of the neck based on SLNB has some limitations. SLNB remains an invasive surgical procedure with associated morbidity. An eventual subsequent completed neck dissection (CND) is a second stage procedure in 30% of patients and should proceed in a timely fashion, which may be a logistic problem. Moreover, subsequent CND is more challenging than elective neck dissection (END) and harbours a higher risk of complications (1).

Although promising results have been reported, the role of FDG-PET/CT to detect occult LNM in HNSCC patients is unclear. Studies are difficult to compare: different scan protocols, definitions of the N0 neck, criteria for PET positivity, ways of reading scans, and reference standards have been used (4). Routine histopathological examination of neck dissection specimens can miss micrometastases, whereas step serial sectioning and immunohistochemistry (as performed in SLNB) can increase the yield by as much as 15.2% (5). Therefore, the most reliable reference standard, which has not been used in PET/CT studies before, is SLNB and follow-up without treatment of the neck in case of negative SLNB (4). Since PET imaging has improved considerably due to technical advances enabling integration of PET and CT devices, improvements in detector capabilities yielding higher image resolution, and optimization of head and neck acquisition parameters, prospective studies are needed (6).

The number of SLNB procedures and second stage CND can be reduced by performing direct neck dissection without SLNB procedures in patients in whom occult LNM can be predicted with very high PPV by FDG-PET/CT. By varying cut-off levels scoring criteria can be developed and optimized to predict the presence of lymph node metastases with high PPV instead of high sensitivity, which is usually done. When focused on high PPV the sensitivity will probably be lower, but missed LNM will then be detected by SLNB when performed after negative FDG-PET/CT.

This proposed prospective multicenter study aims to develop FDG-PET/CT scoring criteria through which occult LNM in patients with early-stage OSCC are detected with high PPV,

reducing the number of SLNB needed. SLNB has a high sensitivity and negative predictive value to detect occult LNM but is invasive. When positive it warrants a second stage neck dissection with a higher risk of complications than when performed at the same stage as resection of the primary tumor. This affects the quality of life. Moreover, it is possible that FDG-PET/CT also finds occult LNM otherwise undetected by SLNB, improving survival.

2. OBJECTIVE(S)

Primary Objective:

The primary objective of this study is to reduce the need for SLNB by FDG-PET/CT in cN0 OSCC patients.

Secondary Objective(s)

The secondary objectives of this study are:

- To optimize scoring criteria for the detection of (occult) lymph node metastases by FDG-PET/CT with a high positive predictive value.
- To assess the sensitivity, specificity, PPV, negative predictive value and accuracy for the different scoring criteria.
- To investigate inter-observer agreement before and after scoring criteria are established.
- To compare the quality of life and costs in three different diagnostic scenarios 1) PET/CT, 2) SLNB, and 3) PET/CT and, only if negative, SLNB.
- To obtain insight into patients' preferences and experience of the diagnostic procedures.

3. STUDY DESIGN

This study is designed as a prospective Dutch multicenter cohort study. A total of 159 patients with early-stage OSCC (cT1-3, cN0, M0), scheduled for transoral tumor resection and SLNB, will be included in a multicenter study to evaluate the potential reduction of SLNB procedures using newly developed FDG-PET/CT scoring criteria and assess the diagnostic accuracy of FDG-PET/CT for the detection of cervical lymph node metastasis in cN0 OSCC patients with and without use of these scoring criteria. The overall study matrix is indicated in Table 1.

Patients with early-stage oral cavity carcinoma (cT1-3, cN0, M0; tumor location will be limited to the intraoral areas of mucosal lip, buccal mucosa, lower alveolar ridge, upper alveolar ridge, retromolar gingiva, retromolar trigone, floor-of-the-mouth, hard palate, and the mobile portion of the oral tongue) scheduled for transoral tumor resection with SLNB will be asked to participate in the study (see Appendix 1: Flowchart regarding the inclusion of patients).

After informed consent is obtained each included patient will undergo FDG-PET/CT and SLNB within a maximum of 3 weeks. It is likely that the majority of the cases, the SLNB and FDG-PET/CT can be performed within a one-week timeframe. Efforts will be made to achieve this timeframe.

The PET/CT will be acquired using dedicated EANM Research Ltd. (EARL2) accredited PET/CT systems (7). Patient preparation, scanner calibration, image acquisition, and reconstruction will be performed according to EANM standards (8,9). After a fasting period of at least 6 hours, patients receive an intravenous injection of FDG. Approximately 60 min after the administration of the tracer a PET-low dose CT of the head and neck and chest (arms down) are acquired. After the PET-low dose CT a contrast-enhanced CT of the head and neck will be performed. When the patient has had a previous allergic reaction after administration of contrast fluid, only a PET-low dose CT of the head and neck and chest is sufficient for participating in this study and a contrast-enhanced CT will not be performed. To avoid false-positive results by cytological puncture, PET/CT has to be performed before ultrasound-guided fine needle aspiration cytology (USgFNAC). Patients with positive USgFNAC will be excluded for further analysis since the neck is not clinically negative. With a prevalence of 27% and a sensitivity of 15.4%, it is expected that 4.2% of patients will have to be excluded for this reason.

.

The results of the PET/CT scan will not be used to alter surgical planning. Institutional nuclear physicians will score the PET/CT scan on potential other tumor sites in the thorax

and upper abdomen. Relevant findings will be reported to the referring physician (e.g. infection or secondary malignancy in the lungs). After the SLNB each PET/CT scan will be individually scored by a panel of 5 experienced nuclear physiciansbefore and after scoring criteria are established. The SLNB will be blinded for the PET/CT scan. All nodes with increased FDG uptake are scored as definitely positive, probably positive, equivocal, or probably negative. If a discrepancy occurs, adjudication will be conducted by a third nuclear physician. These results will be compared with the SLNB results and follow-up as the reference standard.

On histopathological examination of the neck dissection specimen efforts should be made to identify FDG-positive nodes and examine these similar to sentinel nodes. If no neck dissection is performed, the neck will be observed to see of eventually missed metastases become clinically manifest.

The SLNB procedure will be performed according to the practical and consensus guidelines (10-12). Patients will undergo lymphoscintigraphy (including SPECT-CT) after peritumoral injection of [99mTc]Tc-nanocolloid(-ICG), the day before surgery or the day of surgery. Patients with a positive SLNB will undergo a neck dissection. In case of a negative SLNB patients are observed during follow-up according to the national guidelines.

Follow-up will be at least 12 months after SLNB to allow missed occult lymph node metastases to become clinically detectable. It is expected that 80% of the missed lymph node metastases (false negative) will become manifest in the first 12 months (13). Although not included in this research project (because of time limitations), included patients will be asked for participation in a long-term follow-up study, with at least two years follow-up, to assess late regional recurrences and improve the reference standard even further.

In addition, to gain insight into the patients’ preferences and experience with diagnostic modalities, semi-structured interviews will be conducted with patients at the University Medical Center Utrecht and until data saturation has been reached.

Table 1. Imaging and questionnaires schedule for included patients

(Study) Assessment	Day -30 to day -1	Day -21 to day -1	Day 0	3 months	6 months	9 months	12 months	24 months
Demography	x							
Entry Criteria	x							
Medical History	x							
Vital Sign Assessment	x							
Physical Examination	x							
Concomitant Medications/Treatments	x							
Clinical Lab Evaluation	x							
Informed Consent	***x***							
EORTC QLQ-C30		x		x	x		x	
EORTC-QLQ-H&N35		x		x	x		x	
EQ-5D-5L		x		x	x	x	x	
MCQ		x		x	x	x	x	
SDQ		x		x	x	x	x	
PCQ		x		x	x	x	x	
FDG-PET/CT		***x***						
USgFNAC (if decided)		x						
Sentinel lymph node biopsy			x					
End of KWF study							x	
Additional analyses								x
Adverse Event Monitoring		x						

EORTC QLQ-C30, EORTC-QLQ-H&n35, EQ-5D-5L questionnaires pretreatment and 3, 6 and 12 months after treatment routinely asked for fill out for Dutch Head and Neck Audit / DICA

MCQ = Medical Consumption Questionnaire

SDQ = Shoulder Disability Questionnaire

PCQ = Productivity Cost Questionnaire

FDG = FluoroDeoxyGlucose

PET = Positron Emission Tomography

CT = Computed Tomography

USgFNAC = Ultrasound Guided Fine Needle Aspiration Cytology

KWF = Koningin Wilhelmina Fonds voor de Nederlandse Kankerbestrijding

4. STUDY POPULATION

4.1 Population

A total of 159 patients with early-stage oral cavity carcinoma (cT1-3, N0, M0); tumor location will be limited to the intraoral areas of mucosal lip, buccal mucosa, lower alveolar ridge, upper alveolar ridge, retromolar gingiva, retromolar trigone, floor-of-the-mouth, hard palate, or the mobile portion of the oral tongue.

4.2 Inclusion criteria

To be eligible to participate in this study, a subject must meet all of the following criteria:

- Newly diagnosed early-stage OSCC is defined as clinically T1-3, N0 (only when T3 is assessed based on tumor dimensions of >2 cm and ≤4 cm with DOI >10 mm) (see Appendix 2: Tumor Nodal Metastasis (TNM) Staging 8th Edition), without previous treatment of the neck.
- The patient is ≥18 years of age at the time of consent.
- The patient has no palpable lymph nodes in the neck.
- Clinical nodal staging (cN0) has been confirmed by ultrasound, CT, and/or MRI if performed (not mandatory).
- The patient is a candidate for transoral excision and SLNB.
- The patient has provided written informed consent authorization before participating in the study.

4.3 Exclusion criteria

A potential subject who meets any of the following criteria will be excluded from participation in this study:

- The patient has other pathologies than squamous cell carcinoma;
- The patient has recurrent primary OSCC;
- The patient has a history of treatment of the neck (neck dissection and/or radiotherapy);
- The patient has USgFNAC positive for lymph node metastasis;
- The patient has poorly controlled diabetes mellitus;
- The patient refused preoperative imaging workups.

A potential subject who meets the following criteria will be excluded for the contrast-enhanced CT of the head and neck only:

- The patient has had a previous allergic reaction after administration of contrast fluid.

4.4 Sample size calculation

Since it is unlikely that FDG-PET/CT will detect micrometastases with a sensitivity high enough to refrain from (elective) neck dissection if FDG-PET/CT is negative, the focus of this study will be on a PPV high enough to avoid SLNB with second stage neck dissection if FDG-PET/CT is positive. The sample size calculation is made on the number of early-stage OSCC patients, who undergo FDG-PET/CT, needed to reduce reliably the number of (positive) SLNB procedures by 25%.

Unfortunately, no studies with histopathological examination of neck dissection specimens or SLNB and follow-up as reference standard are available which vary cut-off values of objective scoring parameters, e.g. SUVmax, to find a high PPV for the detection of occult lymph node metastases. All such studies focused on a high sensitivity or the combination of sensitivity and specificity, whereas for the present study particularly a high PPV is needed. The study of Peltenburg et al compared SUVmax and lymph node size with the outcome of USgFNAC (14). Although the sensitivity of USgFNAC is limited, its specificity is near 100%. In 52 patients with lymph nodes smaller than 10 mm (radiologically negative) SUVmax was >4.9 in 17 patients of whom 12 had USgFNAC positive lymph nodes. Thus, if patients with SUVmax >4.9 would undergo directly a neck dissection in at least 12 patients a SLNB (with neck dissection as a second stage operation if SLNB is positive) could correctly be avoided. Using this SUVmax cut-off value a reduction of SLNB of 32.7% can be obtained and only a few unnecessary neck dissections will be performed.

To show with an 80% power and a one-sided alpha of 0.05 that the reduction of SLNB of 0.327 is not inferior to 0.25, we need 143 patients. This sample size was calculated using the PASS power software, option: non-inferiority test for one proportion, exact test.
As explained before it is expected that 4.2% of patients have to be excluded because of positive USgFNAC after to FDG-PET/CT. Further drop-out rate for this study is expected to be 5%. After all, generally, patients want to have an extensive diagnostic work-up. FDG-PET/CT is a routine diagnostic examination with a limited burden on the patient. Therefore, a total drop-out rate of (not more than) 10% is expected. Accordingly, this study's population should consist of 159 patients to enable sufficient statistical power.

With the sample size of 143 patients, we expect 47 patients to have a positive FDG-PET/CT scan. Based on the study of Peltenburg et al, we anticipate 33 positive and 14 negative histopathological examination results (14). This results in a PPV of 0.70. Based on binomial exact calculation of the 95% confidence interval (CI), with 47 patients having a positive FDG-PET/CT and a PPV of 0.70, an associated 95% CI of 0.56-0.84 is

expected.

5. TREATMENT OF SUBJECTS

5.1 Investigational product/treatment

The diagnostic intervention of this study is one extra FDG-PET/CT before conventional treatment and questionnaires before treatment and after treatment (at 3, 6, 9, and 12 months).

5.2 Use of co-intervention (if applicable)

After a fasting period of at least 6 hours, patients receive an intravenous injection of FDG. Approximately 60 min after the administration of the tracer a PET-low dose CT of the head and neck and chest abdomen (arms down) is acquired. After the PET-low dose CT a contrast-enhanced CT of the head and neck will be performed. To avoid false-positive results by cytological puncture, PET/CT has to be performed before USgFNAC. Patients with positive USgFNAC will be excluded for further analysis since the neck is not clinically negative.

5.3 Escape medication (if applicable)

Not applicable.

6. INVESTIGATIONAL PRODUCT

Not applicable.

6.1 Name and description of investigational product(s)

6.2 Summary of findings from non-clinical studies

6.3 Summary of findings from clinical studies

6.4 Summary of known and potential risks and benefits

6.5 Description and justification of route of administration and dosage

6.6 Dosages, dosage modifications and method of administration

6.7 Preparation and labelling of Investigational Medicinal Product

6.8 Drug accountability

7. NON-INVESTIGATIONAL PRODUCT

Not applicable.

7.1 Name and description of non-investigational product(s)

7.2 Summary of findings from non-clinical studies

7.3 Summary of findings from clinical studies

7.4 Summary of known and potential risks and benefits

7.5 Description and justification of route of administration and dosage

7.6 Dosages, dosage modifications and method of administration

7.7 Preparation and labelling of Non Investigational Medicinal Product

7.8 Drug accountability

8. METHODS

8.1 Study parameters/endpoints

8.1.1 Main study parameter/endpoint

The main study endpoint is the reduction of (positive) SLNB procedures for detection of (occult) cervical lymph node metastasis in cN0 OSCC patients by FDG-PET/CT.

8.1.2 Secondary study parameters/endpoints (if applicable)

Secondary study endpoints are:

- Results of different scoring criteria, as interpreted by blinded review criteriafor detection of occult lymph node metastases by FDG-PET/CT with a high PPV and accompanying sensitivity, specificity and negative predictive value using different FDG-PET/CT parameters and cut-off values:
    - Lymph nodes with FDG uptake exceeding normal physiological uptake will be considered positive / suspicious for lymph node metastases.
    - Visual scoring: A 5-point Likert scale will be used: 1, completely negative; 2, probably negative; 3, equivocal; 4, probably positive; 5, positive based on the local surrounding background.
    - Images will also be interpreted using the standardized Hopkins criteria. The Hopkins criteria use the internal jugular vein (IJV) and the liver as background blood pool reference (1 = uptake less than IJV, 2 = focal uptake greater than IJV but less than liver, 3 = diffuse uptake greater than IJV or liver, 4 = focal uptake greater than liver, 5 = intense uptake) (15).
    - Semi-quantitative scoring: All nodes with FDG-uptake and/or nodes of 3 mm or larger on PET/CT will be quantified by a manual volume of interest (VOI) drawing around these lymph nodes. SUV calculations are performed using the lean body mass corrected formula. As the maximum SUV value (SUVmax) is susceptible to artefacts such as noise and the SUVmean is observer-dependent, several other SUV metrics are explored, based on specified thresholds of the maximum uptake (SUV50, SUV70, SUV90). In total, 6 different standard uptake values (SUVmean, SUVmax, SUVpeak, SUV50, SUV70, SUV90) are calculated. Lesion-to-background ratios for 2 metrics (SUVmean and SUVmax) based on different background regions are calculated (liver and mediastinal blood pool

for whole body acquisitions and jugular vein, cerebellum, and muscle tissue for dedicated head and neck acquisition) which included ratios based on the liver, mediastinum, cerebellum, and several different muscle regions (16).

  - Morphology: Of lymph nodes of 3 mm or larger the maximal axial diameter, minimal axial diameter, maximum longitudinal/short axis diameter ratio, non-fat low density, and shape (spheric or round) will be scored. These parameters may have additional value in combination with PET parameters.

- Inter-observer agreement between before and after scoring criteria are established;
- Quality of life will be explored with questionnaires. The following questionnaires will be used to measure the quality of life: EORTC QLQ-C30, EORTC-QLQ-H&N35, specific for head and neck cancer patients, EQ-5D-5L, and Shoulder Diability Questionnaire (SDQ) (17-19).
- Costs of different procedures will be collected for every patient arm from the electronic hospital records in every participating hospital and linked to Dutch unit costs. Both the Medical Consumption Questionnaire (MCQ) and the Productivity Cost Questionnaire (PCQ) will be sent to collect costs outside the hospital and productivity losses for each patient. The PCQ is a generic questionnaire designed to determine the costs of productivity losses. In addition, to gain detailed insight into the costs of surgery we aim to perform a microcosting study of SLNB and neck dissection using the Activity Based Costing Method.
- Patients' preferences and experience of the diagnostic procedures will be explored by qualitative research with semi-structured interviews based on an interview guide with a topic list with patients at the University Medical Center Utrecht and until data saturation has been reached.
- Additional (clinically relevant) findings of PET/CT, e.g. distant metastases and second primary tumors, will be scored and the effect on a treatment plan (change yes or no) and intent (curative or palliative) based on multidisciplinary team conference (MDTC) decisions will be analyzed.

8.1.3 Other study parameters (if applicable)

Not applicable.

8.2 Randomisation, blinding and treatment allocation

Not applicable.

8.3 Study procedures

Patients diagnosed with cT1-3N0 OSCC scheduled for SLNB will be identified and screened for eligibility at the weekly multidisciplinary head and neck oncology meeting. If eligible, patients will be informed about this study. After the mandatory waiting period (three days, or more if applicable as long as needed), written informed consent will be obtained from patients and patients will be included.

Table 1. represents the process of participants during the study procedure.

8.4 Withdrawal of individual subjects

Subjects can leave the study at any time for any reason if they wish to do so without any consequences. The investigator can decide to withdraw a subject from the study for urgent medical reasons.

8.4.1 Specific criteria for withdrawal (if applicable)

Not applicable.

8.5 Replacement of individual subjects after withdrawal

After the withdrawal of a subject before surgery, the subject will be replaced to obtain the intended 159 patients in the study group. If the subject withdraws after surgery, data of the subject till withdrawal will be used.

8.6 Follow-up of subjects withdrawn from treatment

If the subject withdraws after surgery, data of the subject till withdrawal will be used.

8.7 Premature termination of the study

In case the study is ended prematurely, the sponsor will notify the accredited METC within 15 days, including the reasons for the premature termination. Criteria for terminating the study prematurely are increased incidence of serious side effects in patients undergoing FDG-PET/CT.

9. SAFETY REPORTING

9.1 Temporary halt for reasons of subject safety

In accordance with section 10, subsection 4, of the WMO, the sponsor will suspend the study if there is sufficient ground that continuation of the study will jeopardize the subject health or safety. The sponsor will notify the accredited METC without undue delay of a temporary halt including the reason for such an action. The study will be suspended pending a further positive decision by the accredited METC. The investigator will take care that all subjects are kept informed.

9.2 AEs, SAEs, and SUSARs

9.2.1 Adverse events (AEs)

Adverse events are defined as any undesirable experience occurring to a subject during the study, whether or not considered related to diagnostic intervention an extra FDG-PET/CT. Adverse events classified according to the CTCAE v.5.0 criteria grade 3 or higher, reported spontaneously by the subject or observed by the investigator or his staff will be recorded. This accounts for all adverse events that occur within the period from FDG intravenous injection until 2 hours after injection. It is not expected that any later occurring events can be attributed to the injection of FDG.

9.2.2 Serious adverse events (SAEs)

A serious adverse event is any untoward medical occurrence or effect that

- results in death;
- is life-threatening (at the time of the event);
- requires hospitalisation or prolongation of existing inpatients' hospitalization;
- results in persistent or significant disability or incapacity;
- is a congenital anomaly or birth defect; or
- any other important medical event that did not result in any of the outcomes listed above due to medical or surgical intervention but could have been based upon appropriate judgment by the investigator.
- an elective hospital admission will not be considered a serious adverse event.

The investigator will report all SAEs to the sponsor without undue delay after obtaining knowledge of the events.

This study is expected to pose a negligible risk for patients; any serious adverse events occurring during 2 hours after injection are expected to be related to the standard treatment and not to the study intervention. Because of this, SAEs that results in death or are life

threatening will not be reported expedited through the web portal *ToetsingOnline* to the accredited METC that approved the protocol. The SAEs will be documented by the investigator and will be reported once a year.

9.2.3 Suspected unexpected serious adverse reactions (SUSARs)

Not applicable.

9.3 Annual safety report

Not applicable.

9.4 Follow-up of adverse events

All AEs will be followed until they have abated, or until a stable situation has been reached. Depending on the event, follow-up may require additional tests or medical procedures as indicated, and/or referral to the general physician or a medical specialist.
SAEs need to be reported till end of study within the Netherlands, as defined in the protocol.

9.5 Data Safety Monitoring Board (DSMB) / Safety Committee

Not applicable.

10. STATISTICAL ANALYSIS

All data is analyzed with professional statistics software (IBM SPSS Statistics Version 26.0). Data are expressed as mean, standard deviations (for parametric continuous variables), and as median and quartiles (for nonparametric continuous variables). The number of cases and percentages are presented as categorical variables. Normal distribution will be verified by using the Shapiro-Wilk test. A p-value of < 0.05 was regarded as statistically significant.

Handling missing Data

Missing data will be handled using multiple imputation strategies. Results across multiple imputation data sets will be combined using the Rubin rules. LASSO-based logistic regression analysis will be used to identify the optimal panel for distinguishing patients with metastases from those without. This method is suitable for regression with a large number of variables and a relatively limited number of events and protects against overfitting. To estimate discriminative value we will compute the area under the curve (AUC) of the Receiving Operator Characteristic Curve (ROC).

10.1 Primary study parameter(s)

Per (newly developed) scoring criteria the reduction of (positive) SNLB procedures will be calculated as well as the number of unnecessary neck dissections (accompanying false positives).

10.2 Secondary study parameter(s)

Accuracy

Analysis of the accuracy of FDG-PET/CT will be performed per patient, per neck side, and neck level (I-V) (as performed by Bae et al (20)). Results of SLNB and follow-up of 12 months will be used as the reference standard.

The most important secondary outcome is the positive predictive value, calculated as the number of positivepatients/necks/levels on FDG-PET/CT with histologically confirmed lymph node metastases divided by the total number of patients/necks/levels with a positive FDG-PET/CT.

In addition, sensitivity, specificity and negative predictive value, and the proportion of true positives and false positives will be estimated.

For estimation of 95% confidence intervals, we will use multistage bootstrap to account for the correlation between sides/levels of necks from the same patients, as was described by Lowe et al (21). We will repeat the statistical analysis for a scenario where in clinical practice all positive FDG-PET/CTs would not be followed by SLNB but a neck

dissection at the same operation as the resection of the primary tumor. Such a scenario could be useful to reduce the number of SLNBs and second stage neck dissections.

FDG-PET/CT scoring parameters

Models based on combinations of FDG-PET/CT parameters will be constructed and analysed for accuracy. The accuracy of scoring parameters will be analysed for a wide range of cut-off values for each parameter, through which sensitivity, specificity, positive predictive value, and negative predictive value will vary, allowing for choosing the optimal results for the specific aim of this study: a high positive predictive value (PPV) to reduce the number of SLNBs with acceptable sensitivity.

We will first compare the different scoring results (visual, semi-quantitative) and the morphology characteristics of the patients with and without metastases (based on the reference standard of SLNB and/or 12-month follow-up). Comparison of continuous data will be expressed as differences in mean ± standard deviation (SD) when normally distributed, and as median ± interquartile range (IQR) when skewed. Univariate analysis using independent samples T-tests will be applied for parametric continuous variables, while Friedman's 2-way ANOVA's and Wilcoxon signed rank tests will be applied for non-parametric continuous variables. Comparison of categorical variables will be expressed as differences in proportions. For comparing categorical data $\chi 2$-tests will be used.

Searching for the optimal cut-off values and subsequently assessing performance at these cut-off values within the same dataset can result in an overly optimistic estimation of performance as described by Leeflang et al (22).

Interobserver agreement

The panel of observers will consist of 5 experienced nuclear medicine physicians from participating centers. For all scoring criteria and newly developed scoring models interobserver agreement will be assessed. Concordance in measurements and scoring between observers are calculated using a repeated measures ANOVA. Agreement between different observers is assessed by calculating Fleiss' kappa (κ) and intraclass correlation coefficients (ICCs) and accompanying 95% confidence intervals using a two-way mixed single measures model with absolute agreement. The κ values are graded as slight (0.01 – 0.20), fair (0.21-0.40), moderate (0.41-0.60), substantial (0.61-0.80) or almost perfect (0.81-0.99) agreement [26]. The ICCs are rated as poor (0.00 – 0.49), fair to good (0.50 – 0.74) and excellent (0.75 – 1.00). Bland-Altman plots are constructed to visualize agreement.

Cost-effectiveness analysis

We aim to calculate total costs for the patients included in the study. In addition, we aim to perform scenario analysis in which we calculate the impact of diagnosing and subsequent treatment of the patient based on the newly developed scoring criteria. In model based approaches three different diagnostic scenarios will be compared concerning quality of life and costs: 1) PET/CT, 2) SNLB and 3) PET/CT and, only if negative, SNLB. These models are based on PET/CT and SNLB results. Using costing data from our cohort and literature input we aim to draft scenario's analysing costs for True Positive (TP), True Negative (TN), False Positive (FP) and False Negative (FN). See figure 1. Also incremental cost-effectiveness ratios for the different scenarios will be calculated.

Extensive (probabilistic and deterministic) sensitivity analyses will be performed. The analyses will be performed according the Dutch guidelines for economic evaluations in healthcare, taking into account different levels in costs per unit and health care resources that contributed most to the total costs difference per group. Results are shown in a cost-effectiveness plane to obtain full insight in outcomes.

We expect an impact on both costs and effects outside the study period, and will therefore combine costs and effects of the trial with long-term effect outcomes obtained from literature in a decision-analytic model (Markov) to estimate future costs for these patients.

Sensitivity analysis

Extensive (probabilistic and deterministic) sensitivity analyses will be performed. The analyses will be performed according the Dutch guidelines for economic evaluations in healthcare, taking into account different levels in costs per unit and health care resources that contributed most to the total costs difference per group. Results are shown in a cost-effectiveness plane to obtain full insight in outcomes.

Patient outcome analysis

Quality of life will be measured using the EQ-5D questionnaire, which consists of five items measuring problems on five dimension of quality of life (mobility, self-care, usual activities, pain/discomfort and anxiety/depression). The resulting profile of answers can be transformed to a utility given by the general public: the EQ-5D-5L index using the Dutch index tariff. Questionnaires will be send out at baseline, 3, 6, 9 and 12 months.

The shoulder disability questionnaire (SDQ) is a validated pain-related disability questionnaire including 16 items that describe common conditions that may induce symptoms in patients with disorders of the shoulder. All items refer to the preceding 24 hours. Options are “yes”, “no” and “not applicable”. The “not applicable” category should be used when the condition referred to has not occurred during the preceding 24 hours. A final score is calculated dividing the number of “yes” scored items by the total number of items applicable. And then multiplying the score by 100 results in a final score that ranges between 0 (no disability) and 100 (all applicable items scored “yes”). The higher the score, the greater the impairment was. All patients are asked to fill out the questionnaires for both the left and the right shoulder separately (23). Questionnaires will be send out at baseline, 3, 6, 9 and 12 months postoperative. Multiple logistic regression is performed to assess which variables predicted an SDQ-score >0.

Patients’ preference and experience

Finally to obtain insight in the patients’ preference and experience procedures semi-structured interviews will be analysed by two researchers using thematic descriptive analyses. This thematic analysis will be an independent qualitative descriptive approach to identify, analyse and report patterns (themes) within the data provided by the semi-structured interviews and permits us as research team to combine analysis of meaning within their particular context. During analyses we will search for the identification of common threads that extend across the interviews. This will provide a detailed, and nuanced account of data by breaking the interview texts into relatively small units. Practically the semi-structured interviews will be transcribed, and will be thoroughly read several times. Thereafter initial codes will be generated, followed by the search for themes, reviewing these themes and finally defining and naming the themes. These themes will be reported and will be supported by compelling extract examples relating back to the analysis to answer the research question. Semi-structured interviews will be analyzed by two researchers using thematic descriptive analyses (24). Data coding will be done by open, axial and selective coding and will be supported by the software package NVivo. Patients for semi-structured interviews will only be included at the University Medical Center Utrecht and until data saturation has been reached.

10.3 Other study parameters

Not applicable.

10.4 Interim analysis (if applicable)

Not applicable.

11. ETHICAL CONSIDERATIONS

11.1 Regulation statement

This study will be conducted according to the principles of the Declaration of Helsinki (October 2013) and in accordance with the Medical Research Involving Human Subjects Act (in Dutch: WMO) and other guidelines, regulations and acts.

11.2 Recruitment and consent

Patients will be informed briefly by their head and neck surgeon about the study when patients fulfil the inclusion criteria. This will happen during the outpatient clinic consultation following the multidisciplinary meeting of the head and neck working group. Afterwards, the concerning head and neck surgeon will ask the patient whether he/she gives consent to be approached by the executive researcher of this study to provide more information about this study.

When patients consent to being further informed about this study by the executive researcher, the executive researcher will inform the patient about the research. Consequently, patients will receive the patient information folder regarding this study and will be asked whether they consent to being called by telephone three days later, or longer if necessary, by the executive researcher, providing enough time for patients to evaluate whether they would want to participate in this study.

If consent for a telephonic approach has been given, patients will be called by telephone if they have questions regarding this study and for their provisional informed consent. This way patients do not have to pay an extra visit to the hospital to give their informed consent regarding participation in this study, while still enabling to schedule FDG-PET/CT for the concerning patient.

Even though FDG-PET/CT will be scheduled for the patient after provisional informed consent, patients are still able to end their participation in this study at any time without further explanation.

Finally, patients will be asked, by the executive researcher, to sign the informed consent in duplicate on the day of their first study-related examination (being the day of FDG-PET/CT).

11.3 Objection by minors or incapacitated subjects (if applicable)

Not applicable for this study. Minors and incapacitated will be excluded as described in section 9.4.

11.4 Benefits and risks assessment, group relatedness

Benefits

The SLNB procedure is standard care for these early-stage OSCC patients in the participating centers. These patients usually do not undergo FDG-PET/CT. An extra FDG-PET/CT may detect other tumor sites in the thorax or upper abdomen. Patients may benefit from this. FDG-PET/CT is considered a safe procedure, with limited radiation exposure.

Risks

False-positive findings of FDG-PET/CT may give burden to the patients. This may include other additional examinations of the thorax and/or upper abdomen to confirm PET/CT findings. FDG-PET/CT is considered a safe procedure, with limited radiation exposure. Therefore, we conclude the risk is negligible for this study according to the NFU guidelines.

11.5 Compensation for injury

The sponsor/investigator has a liability insurance which is in accordance with article 7 of the WMO.

The sponsor (also) has an insurance which is in accordance with the legal requirements in the Netherlands (Article 7 WMO). This insurance provides cover for damage to research subjects through injury or death caused by the study.
The insurance applies to the damage that becomes apparent during the study or within 4 years after the end of the study.

11.6 Incentives (if applicable)

There are no financial incentives applicable for participants of this study. Participant will be eligible for reimbursement of travel costs, only if additional hospital visits are needed for this study, i.e. if the extra FDG-PET/CT cannot be combined with a regular hospital visit.

12. ADMINISTRATIVE ASPECTS, MONITORING AND PUBLICATION

12.1 Handling and storage of data and documents

After the informed consent is signed, each patient receives a unique study number and 3-letter code. The key to the patients name and the other identifying characteristics is safeguarded by the investigator and PI, who is the only one with access to the source data. Data will be kept for 15 years. Data management will be prepared by the investigator in cooperation with on-site data managers. A web-based data management system (Castor EDC) will be used for data management. The subject's privacy is protected by the usage of the study number, instead of personal data. For detailed information regarding the handling and storage of data, we refer to the enclosed 'Datamanagement Plan'.

12.2 Monitoring and Quality Assurance

This study is classified as negligible risk because of the safety of the non-investigational products with a low risk on mild damage for the subjects. Therefore monitoring should take place by a Julius clinical for optimal quality assurance once during the duration of the study according to the NFU guidelines.
For detailed information regarding monitoring and quality assurance, we refer to the enclosed 'Monitoring plan'.

12.3 Amendments

Amendments are changes made to the research after a favourable opinion by the accredited METC has been given. All amendments will be notified to the METC that gave a favourable opinion.

All substantial amendments will be notified to the METC.

Non-substantial amendments will not be notified to the accredited METC and the competent authority, but will be recorded and filed by the sponsor.

12.4 Annual progress report

The sponsor/investigator will submit a summary of the progress of the trial to the accredited METC once a year. Information will be provided on the date of inclusion of the first subject, numbers of subjects included and numbers of subjects that have completed the trial, serious adverse events/ serious adverse reactions, other problems, and amendments.

12.5 Temporary halt and (prematurely) end of study report

The investigator/sponsor will notify the accredited METC of the end of the study within a period of 8 weeks. The end of the study is defined as the last patient's last visit.

The sponsor will notify the METC immediately of a temporary halt of the study, including the reason of such an action.

In case the study is ended prematurely, the sponsor will notify the accredited METC within 15 days, including the reasons for the premature termination.

Within one year after the end of the study, the investigator/sponsor will submit a final study report with the results of the study, including any publications/abstracts of the study, to the accredited METC.

12.6 Public disclosure and publication policy

The results (positive or negative) of this study will be disclosed unreservedly. Data and results of research are owned by the investigators. The results of research will be submitted for publication to peer-reviewed scientific journals. Disputes on the interpretation of the results may not lead to an unnecessary delay in publication. None of the parties concerned has a right of veto. The parties concerned must attempt to resolve disputes by negotiation. Should one of the parties feel that it has been disadvantaged, or should any other problem relating to publication arise, the parties can contact the medical ethics committee for mediation.

13. STRUCTURED RISK ANALYSIS

Not applicable.

13.1 Potential issues of concern

13.2 Synthesis

14. REFERENCES

1. de Bree R, de Keizer B, Civantos FJ, Takes RP, Rodrigo JP, Hernandez-Prera JC, Halmos GB, Rinaldo A, Ferlito A. What is the role of sentinel lymph node biopsy in the management of oral cancer in 2020? Eur Arch Otorhinolaryngol. 2021 Sep;278(9):3181-3191.
2. Liu M, Wang SJ, Yang X, Peng H. Diagnostic Efficacy of Sentinel Lymph Node Biopsy in Early Oral Squamous Cell Carcinoma: A Meta-Analysis of 66 Studies. PLoS One 2017; 12:e0170322.
3. Kim DH, Kim Y, Kim SW, Hwang SH. Usefulness of Sentinel Lymph Node Biopsy for Oral Cancer: A Systematic Review and Meta-Analysis. Laryngoscope. 2021 Feb;131(2):E459-E465.
4. de Bree R, Hoekstra OS. The potential of FDG-PET in the detection of occult lymph node metastasis: importance of patient selection and reference standard. Eur Arch Otorhinolaryngol. 2013 Aug;270(8):2173-4.
5. Rinaldo A, Devaney KO, Ferlito A. Immunohistochemical studies in the identification of lymph node micrometastases in patients with squamous cell carcinoma of the head and neck. ORL J Otorhinolaryngol Relat Spec. 2004; 66(1):38-41.
6. Ferris RL, Cramer JD, Branstetter Iv BF. Positron Emission Tomography/Computed Tomography in Evaluation of the Clinically N0 Neck in Head and Neck Squamous Cell Carcinoma. J Clin Oncol. 2019 Jul 10;37(20):1683-1685.
7. Kaalep A, Sera T, Oyen W, Krause BJ, Chiti A, Liu Y, Boellaard R. EANM/EARL FDG-PET/CT accreditation - summary results from the first 200 accredited imaging systems. Eur J Nucl Med Mol Imaging. 2018;45:412-422.
8. Boellaard R, Delgado-Bolton R, Oyen WJ, Giammarile F, Tatsch K, Eschner W, Verzijlbergen FJ, Barrington SF, Pike LC, Weber WA, Stroobants S, Delbeke D, Donohoe KJ, Holbrook S, Graham MM, Testanera G, Hoekstra OS, Zijlstra J, Visser E, Hoekstra CJ, Pruim J, Willemsen A, Arends B, Kotzerke J, Bockisch A, Beyer T, Chiti A, Krause BJ; European Association of Nuclear Medicine (EANM). FDG PET/CT: EANM procedure guidelines for tumour imaging: version 2.0. Eur J Nucl Med Mol Imaging. 2015 Feb;42(2):328-54.
9. Kaalep A, Sera T, Rijnsdorp S, Yaqub M, Talsma A, Lodge MA, Boellaard R. Feasibility of state of the art PET/CT systems performance harmonisation. Eur J Nucl Med Mol Imaging. 2018 Jul; 45(8):1344-1361.
10. Alkureishi LW, Burak Z, Alvarez JA, Ballinger J, Bilde A, Britten AJ, Calabrese L, Chiesa C, Chiti A, de Bree R, Gray HW, Hunter K, Kovacs AF, Lassmann M, Leemans CR, Mamelle G, McGurk M, Mortensen J, Poli T, Shoaib T, Sloan P,

Sorensen JA, Stoeckli SJ, Thomsen JB, Trifiro G, Werner J, Ross GL; European Association of Nuclear Medicine Oncology Committee; European Sentinel Node Biopsy Trial Committee. Joint practice guidelines for radionuclide lymphoscintigraphy for sentinel node localization in oral/oropharyngeal squamous cell carcinoma. Ann Surg Oncol. 2009 Nov;16(11):3190-210.

11. Schilling C, Stoeckli SJ, Vigili MG, de Bree R, Lai SY, Alvarez J, Christensen A, Cognetti DM, D'Cruz AK, Frerich B, Garrel R, Kohno N, Klop WM, Kerawala C, Lawson G, McMahon J, Sassoon I, Shaw RJ, Tvedskov JF, von Buchwald C, McGurk M. Surgical consensus guidelines on sentinel node biopsy (SNB) in patients with oral cancer. Head Neck. 2019 Aug; 41(8):2655-2664.
12. Giammarile F, Schilling C, Gnanasegaran G, Bal C, Oyen WJG, Rubello D, Schwarz T, Tartaglione G, Miller RN, Paez D, van Leeuwen FWB, Valdés Olmos RA, McGurk M, Delgado Bolton RC. The EANM practical guidelines for sentinel lymph node localisation in oral cavity squamous cell carcinoma. Eur J Nucl Med Mol Imaging. 2019 Mar; 46(3):623-637.
13. Flach GB, Tenhagen M, de Bree R, Brakenhoff RH, van der Waal I, Bloemena E, Kuik DJ, Castelijns JA, Leemans CR. Outcome of patients with early stage oral cancer managed by an observation strategy towards the N0 neck using ultrasound guided fine needle aspiration cytology: No survival difference as compared to elective neck dissection. Oral Oncol. 2013 Feb; 49(2):157-64.
14. Peltenburg B, de Keizer B, Dankbaar JW, de Boer M, Willems SM, Philippens MEP, Terhaard CHJ, de Bree R. Prediction of ultrasound guided fine needle aspiration cytology results by FDG PET-CT for lymph node metastases in head and neck squamous cell carcinoma patients. Acta Oncol. 2018 Dec; 57(12): 1687-1692.
15. Marcus C, Ciarallo A, Tahari AK, Mena E, Koch W, Wahl RL, Kiess AP, Kang H, Subramaniam RM. Head and neck PET/CT: therapy response interpretation criteria (Hopkins Criteria)-interreader reliability, accuracy, and survival outcomes. J Nucl Med. 2014 Sep;55(9):1411-6.
16. Helsen N, Van den Wyngaert T, Carp L, De Bree R, VanderVeken OM, De Geeter F, Maes A, Cambier JP, Spaepen K, Martens M, Hakim S, Beels L, Hoekstra OS, Van den Weyngaert D, Stroobants S; ECLYPS Consortium, Van Laer C, Specenier P, Maes A, Debruyne P, Hutsebaut I, Van Dinter J, Homans F, Goethals L, Lenssen O, Deben K. Quantification of 18Ffluorodeoxyglucose Uptake to Detect Residual Nodal Disease in Locally Advanced Head and Neck Squamous Cell Carcinoma After Chemoradiotherapy: Results From the ECLYPS Study. Eur J Nucl Med Mol Imaging. 2020 May;47(5):1075-1082.

17. Aaronson NK, Ahmedzai S, Bergman B, Bullinger M, Cull A, Duez NJ, Filiberti A, Flechtner H, Fleishman SB, de Haes JC. The European Organization for Research and Treatment of Cancer QLQ-C30: a quality-of-life instrument for use in international clinical trials in oncology. J Natl Cancer Inst 1993 Mar 3;85(5):365-76.14304 / Remco Bree 2021-10-13 14:57:59 Page 50 of 53
18. Sherman AC, Simonton S, Adams DC, Vural E, Owens B, Hanna E. Assessing quality of life in patients with head and neck cancer: cross-validation of the European Organization for Research and Treatment of Cancer (EORTC) Quality of Life Head and Neck module (QLQ-H&N35). Arch Otolaryngol Head Neck Surg 2000 Apr;126(4):459-67.
19. The EuroQol group. EuroQol–a new facility for the measurement of health-related quality of life. Health Policy 1990; 16:199–208.
20. Bae MR, Roh JL, Kim JS, Lee JH, Cho KJ, Choi SH, Nam SY, Kim SY. 18 F-FDG PET/CT versus CT/MR imaging for detection of neck lymph node metastasis in palpably node-negative oral cavity cancer. J Cancer Res Clin Oncol. 2020 Jan;146(1):237-244.
21. Lowe VJ, Duan F, Subramaniam RM, Sicks JD, Romanoff J, Bartel T, Yu JQM, Nussenbaum B, Richmon J, Arnold CD, Cognetti D, Stack BC Jr. Multicenter Trial of [18 F]fluorodeoxyglucose Positron Emission Tomography/Computed Tomography Staging of Head and Neck Cancer and Negative Predictive Value and Surgical Impact in the N0 Neck: Results From ACRIN 6685. J Clin Oncol. 2019 Jul 10;37(20):1704-1712.
22. Leeflang MM, Moons GM, Zwinderman AH, Reitsma J. Bias in sensitivity and specificity caused by data-driven selection of optimal cutoff values: mechanisms, magnitude, and solutions. Clin Chem. 2008 Apr; 54(4): 729-37.
23. Vaismoradi M, Turunen H, Bondas T. Content analysis and thematic analysis: Implications for conducting a qualitativedescriptive study. Nurs Health Sci. 2013 Sep;15(3):398-405.
24. Brands MT, Campschroer G, Merkx MAW, Verbeek ALM, van Dijk BAC, Geurts SME. Second primary tumours after squamous cell carcinoma of the oral cavity. Eur J Surg Oncol. 2021 Aug; 47(8):1934-1939.

APPENDIX 1 – FLOWCHART REGARDING INCLUSION OF PATIENTS

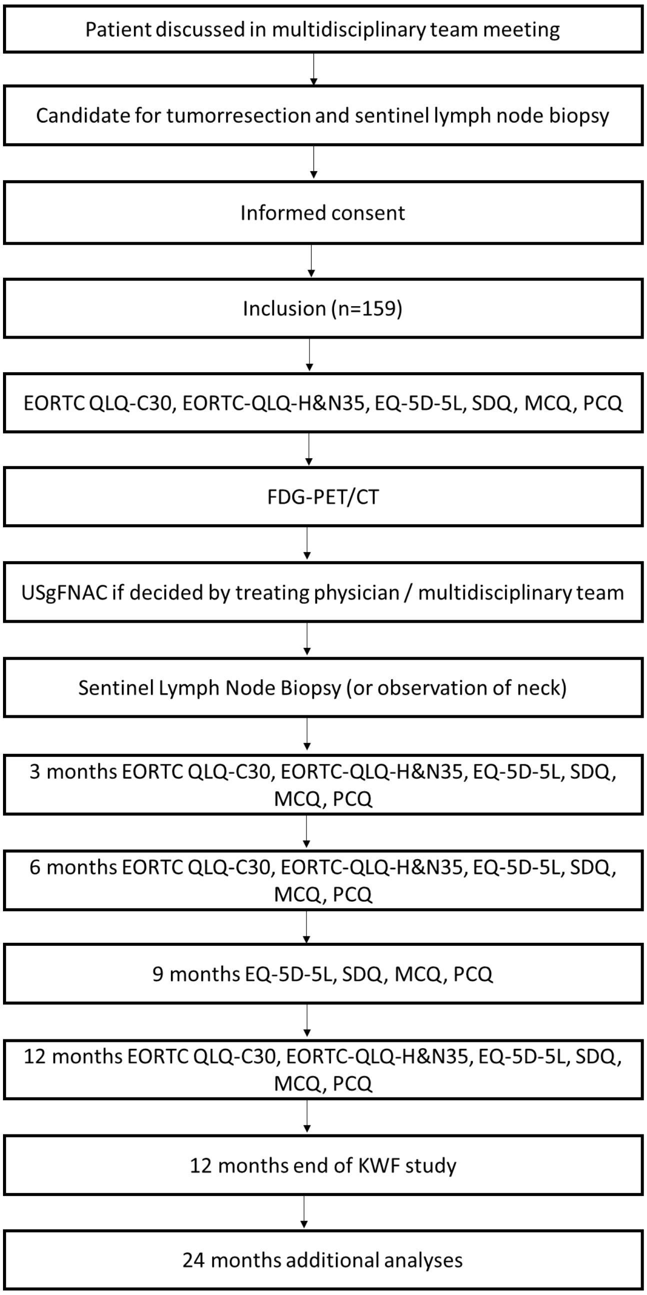

EORTC QLQ-C30, EORTC-QLQ-H&n35, EQ-5D-5L questionnaires pretreatment and 3, 6 and 12 months after treatment routinely asked for fill out for Dutch Head and Neck Audit / DICA

SDQ = Shoulder Disability Questionaire

MCQ = Medical Consumption Questionnaire

PCQ = Productivity Cost Questionnaire

FDG = FluoroDeoxyGlucose

PET = Positron Emission Tomography

CT = Computed Tomography

USgFNAC = Ultrasound Guided Fine Needle Aspiration Cytology

KWF = Koningin Wilhelmina Fonds voor de Nederlandse Kankerbestrijding

APPENDIX 2 – TNM STAGING 8TH EDITION

Oral Cavity Squamous Cell Carcinoma	
**Grade**	**Description**
**Primary Tumor (T)**	
TX	Primary tumor cannot be assessed
T0	No evidence of primary tumor
Tis	Carcinoma in situ
T1	Tumor ≤2 cm with depth of invasion (DOI) ≤5 mm
T2	Tumor ≤2 cm, with DOI >5 mm and ≤10 mm; **or**<br>Tumor >2 cm and ≤4 cm, with DOI ≤10 mm
T3	Tumor >2 cm and ≤4 cm with DOI >10 mm; **or**<br>Tumor >4 cm with DOI ≤10 mm
T4	Moderately advanced or very advanced local disease
T4a	Moderately advanced local disease.<br>Tumor >4 cm with DOI >10 mm; **or**<br>Tumor invades adjacent structures only (e.g., through cortical bone of the mandible or maxilla, or involves the maxillary sinus or skin of the face).<br>NOTE: Superficial erosion of bone/tooth socket (alone) by a gingival primary is not sufficient to classify a tumor as T4.
T4b	Very advanced local disease.<br>Tumor invades masticator space, pterygoid plates, or skull base and/or encases the internal carotid artery.
**Lymph Node (N)**	
NX	Regional lymph nodes cannot be assessed
N0	No regional lymph node metastasis
N1	Metastasis in a single ipsilateral lymph node, 3 cm or smaller in greatest dimension and no extra nodal extension (ENE)
N2	Metastasis in a single ipsilateral node larger than 3 cm but not larger than 6 cm in greatest dimension and ENE(–); **or**<br>Metastases in multiple ipsilateral lymph nodes, none larger than 6 cm in greatest dimension and ENE(–); **or**<br>In bilateral or contralateral lymph nodes, none larger than 6 cm in greatest dimension, and ENE(–).
N2a	Metastasis in a single ipsilateral node larger than 3 cm but not larger than 6 cm in greatest dimension, and ENE(–).
N2b	Metastases in multiple ipsilateral nodes, none larger than 6 cm in greatest dimension, and ENE(–)
N2c	Metastases in bilateral or contralateral lymph nodes, none larger than 6 cm in greatest dimension, and ENE(–)
N3	Metastasis in a lymph node larger than 6 cm in greatest dimension and ENE(–); **or**<br>Metastasis in any node(s) and clinically overt ENE(+)
N3a	Metastasis in a lymph node larger than 6 cm in greatest dimension and ENE(–)
N3b	Metastasis in any node(s) and clinically overt ENE(+)

Distance Metastasis (M)	
Mx	Distant metastasis cannot be assessed
M0	No distant metastasis
M1	Distant metastasis
